# ANGPTL3 accelerates atherosclerotic progression via direct regulation of M1 macrophage activation in plaque

**DOI:** 10.1016/j.jare.2024.05.011

**Published:** 2024-05-11

**Authors:** Yuejie Zhang, Cen Yan, Yuan Dong, Jiwei Zhao, Xuanyi Yang, Yalan Deng, Li Su, Jiming Yin, Yang Zhang, Fenghui Sun, Yingmei Feng

**Affiliations:** a Beijing Institute of Hepatology, Beijing Youan Hospital, Beijing 100069, China; b Department of Science and Technology, Beijing Youan Hospital, Beijing 100069, China; c Neuroscience Research Institute, Peking University Center of Medical and Health Analysis, Peking University, Beijing 100191, China; d Department of Neurology, Beijing Youan Hospital, Beijing 100069, China

**Keywords:** ANGPTL3, Macrophages, Toll-like receptor (TLR)4, NF-κB, Atherosclerosis

## Abstract

**Introduction:**

The N-terminal domain of angiopoietin-like protein 3 (ANGPTL3) inhibits lipoprotein lipase activity. Its C-terminal fibrinogen-like (FBN) domain is a ligand of macrophage integrin αvβ3.

**Objectives:**

ANGPTL3 might home to plaque where it directly regulates macrophage function via integrin αvβ3 for atherosclerosis progression.

**Methods:**

*Ldlr*^-/-^ mice on a high-fat diet and *ApoE*^-/-^ mice on a chow diet were received adeno-associated virus (AAV)-mediated *Angptl3* gene transfer and followed up for 12 weeks. *ApoE*^-/-^ mice were injected AAV containing FLAG-tagged *Angptl3* cDNA for tracing. Atherosclerotic features were compared between *Angptl3*^-/-^*ApoE*^-/-^ mice and *ApoE*^-/-^ littermates. THP-1 cells were exposed to 0 or 50 μg/ml ANGPTL3 FBN domain for 24 h to evaluate Toll-like receptor (TLR)4 expression using western blot analysis and circulating cytokine and chemokine profiles by the MILLIPLEX MAP assay. Phospho-proteomic profile was established in ANGPTL3-treated macrophages. Integrin β3 deficient THP-1 cells were obtained by sgRNAs targeting RGD sequence using Lentivirus-Cas9 system.

**Results:**

*Angptl3* overexpression increased atherosclerotic progression and CD68^+^ macrophages in plaque (p < 0.05 for all). By immunostaining, FLAG^+^ cells were identified in plaque of gene transferred *ApoE*^-/-^ mice. Fluorescent immunostaining detected co-localisation of Angptl3 and CD68 in plaque macrophages. Phospho-proteomic analysis revealed that Angptl3 induced phosphorylation of proteins that were involved in the IL-17 signalling pathway in THP-1 cells. *In vitro*, ANGPTL3 treatment increased the production of interleukin (IL)-1β and tumour necrosis factor-α in THP-1 cells (p < 0.05 for both). Exposure of ANGPTL3 to THP-1 cells induced Akt phosphorylation which was weakened in integrin β3 deficient ones. ANGPTL3 elevated TLR4 expression via Akt phosphorylation. In response to lipopolysaccharide, nuclear factor-κB activity was 2.2-fold higher in THP-1 cells pre-treated with ANGPTL3 than in untreated cells (p < 0.05).

**Conclusions:**

Targeting ANGPTL3 could yield a dual benefit of lowering lipid levels in the blood and suppressing macrophage activation in plaque.

## Highlights

• Hepatic ANGPTL3 overexpression reinforced atherosclerotic progression.
• ANGPTL3 homed to plaque where it directly regulates macrophage function via integrin αvβ3.
• ANGPTL3 enhanced NF-kB activity and inflammatory cytokine production and in THP-1 cells.
• Angptl3 promoted TLR4 expression via Akt phosphorylation.
• ANGPTL3 was present in human atherosclerotic plaque.

## Graphical abstract

Upon secreted into blood, ANGPTL3 homes to plaque via interaction with integrin αvβ3 on macrophages. ANGPTL3 regulates TLR4 protein expression, inflammatory cytokine production such like IL-1β and TNF-α and NF-kB activation for M1 macrophage polarization, leading to plaque progression.

**Figure:**
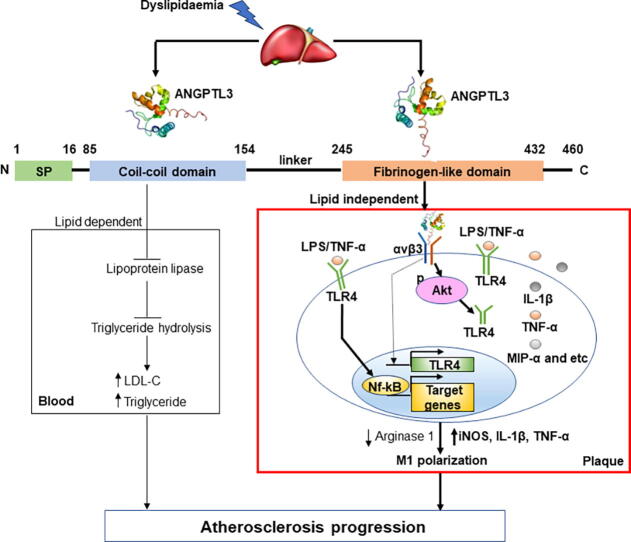

## Introduction

Lipid-lowering drugs such as statins and PCSK9 monoclonal antibodies significantly reduce low-density lipoprotein cholesterol (LDL-c) and the incidence of major adverse cardiovascular events (MACE) [1], [2]. However, when LDL-c levels are decreased to 50 mg/dL, statins fail to prevent the occurrence of MACE [3]. Likewise, the anti-inflammatory therapies such as the interleukin (IL)-1β monoclonal antibody (mAb) canakinumab and colchicine significantly reduced MACE, but had no effect on lipid levels when compared to placebo groups [4], [5]. Notably, under dyslipidaemia, haematopoietic stem/progenitor cells become active and produce inflammatory myeloid cells [6]. Therefore, how to control both dyslipidaemia and inflammation using one drug is a research area of great interest.

Recently, angiopoietin-like protein 3 (ANGPTL3) has emerged as a novel lipid-lowering target. The coiled-coil-domain (CCD) on its N-terminus suppresses the lipoprotein lipase activity that modulates triglyceride hydrolysis. Injection of the anti-ANGPTL3 mAb evinacumab or an *ANGPTL3* antisense oligonucleotide was found to attenuate ANGPTL3 production and potently reduced LDL-c and triglyceride levels in patients with familial hypercholesterolaemia and hypertriglyceridaemia [7], [8]. Interestingly, its C-terminus harbours a fibrinogen-like (FBN) domain, which is a ligand of integrin αvβ3. Interaction between the FBN domain and integrin αvβ3 promotes phosphorylation pathways to regulate endothelial cell proliferation and migration [9]. Given that integrin αvβ3 is also reportedly expressed on macrophages [10], we hypothesised that ANGPTL3 might exhibit plaque homing via binding to integrin αvβ3 to directly regulate certain properties of macrophages involved in atherosclerosis progression.

To test this hypothesis, we evaluated the effect of Angptl3 on atherosclerosis in *Ldlr*^-/-^ mice on a high-fat diet (HFD) and *ApoE*^-/-^ mice on a chow diet, both of which were administered AAV-mediated vector or *Angptl3* gene transfer. To explore Angptl3 homing, *ApoE*^-/-^ mice were injected with AAV-mediated FLAG-tagged *Angptl3* gene transfer, followed by immunostaining of FLAG in plaque. Co-localisation of Angptl3 and CD68 was studied by fluorescent immunostaining. The atherosclerotic features of plaque size and macrophage number were compared between *ApoE*^-/-^ and *Angptl3*^-/-^*ApoE*^-/-^ (DKO) mice. Inflammatory properties were investigated in THP-1 cells exposed to the FBN domain of Angptl3. A comprehensive protein phosphorylation profile was established in ANGPTL3-stimulated THP-1 cells by phospho-proteomics and proteomics.

## Methods

### Human subjects

Five patients with coronary heart disease were enrolled in the department of Neurology in Beijing Youan hospital from November 1st 2023 till December 30th 2023. The plaque in the cerebrovascular artery was carefully dissected out using bare wire thrombectomy technique under the guidance of angiography.

### Mice and treatment

In total, 182 male, wild-type, *Ldlr*^-/-^, *ApoE*^-/-^, and DKO mice on the C57BL/6J background were used in the study. For the DKO, exons 1–7 (aa 1–455) in *Angptl3* was completely deleted using CRISPR-cas9 technology. After obtaining stable *Angptl3*^-/-^ mice, they were crossed with *ApoE*^-/-^ mice for more than four generations to create a stable DKO strain. Age-matched *ApoE*^-/-^ mice served as controls for the DKO strain. To study the effects of Angptl3 on atherosclerotic progression, *Ldlr*^-/-^ mice (age, 8 weeks) and *ApoE*^-/-^ mice (age, 23–25 weeks) were administered AAV carrying *Angptl3* cDNA or empty vector via tail vein injection (1 × 10^11^ viral genomes per mouse). Immediately after injection, the *Ldlr*^-/-^ mice were placed on a HFD (1.25 % cholesterol, 40 % fat) for 12 weeks, and the *ApoE*^-/-^ mice were maintained on a chow diet for 12 weeks.

### Ethics statement

All experiments involving animals were conducted according to the ethical policies and procedures approved by the ethic committee of Capital Medical University (Approval no. AEEI-2023–234). The human study protocol was approved by the competent Institutional review Boards of Beijing Youan hospital (Approval no. LL-2023-146-K). All patients provided written informed consent.

### Statistics

For all experiments, unpaired, two-tailed Student’s tests and Mann-Whitney tests were used to compare the means between two groups. For more than two experimental groups, one-way analysis of variance with Dunnett’s multiple comparison test was applied. Significance was indicated by a two-tailed α-level of 0.05.

Detailed methods are described in the Supplemental Material.

## Results

### Hepatic overexpression of *Angptl3* increased cholesterol levels and plaque progression in hypercholesterolaemia *Ldlr^-^*^/-^ mice

We first evaluated Angptl3 expression in murine atherosclerotic models. Western blotting results showed that hepatic Angptl3 expression was 1.7-fold higher in the *Ldlr*^-/-^ HFD group than in the chow diet group (Fig. S1A). To explore the effect of Angptl3 on atherosclerosis, *Ldlr*^-/-^ mice were injected with AAV-derived vector or mouse *Angptl3* cDNA. Immediately after gene transfer, the mice were placed on HFD for 12 weeks to induce hyperlipidaemia and atherosclerosis. Twelve weeks after gene transfer, qPCR analysis verified EGFP expression in the vector and AAV-ANGPTL3 groups, but not in the non-injected control group, indicating successful gene transfer (Fig. S1B).

After 12 weeks of HFD, hepatic *Angptl3* gene transfer induced a 1.3-fold increase in mRNA expression of *Angptl3* compared with that in the vector group, but the difference did not reach statistical significance (p = 0.11, n = 6–10). Because protein levels of Angptl3 did not differ between non-injected mice and vector-injected mice (p ≥ 0.57), the two groups were pooled in the entire study. Hepatic and blood levels of Angptl3, quantified by ELISA and western blotting, respectively, were comparable between the *Angptl3* gene transferred group and the pooled control group (blood Angptl3: 18.4 ± 6.7 *vs*. 19.8 ± 4.9 ng/mL, respectively, p = 0.45, n = 10–15; and relative hepatic Angptl3: 1.1 ± 0.3 *vs*. 1.2 ± 0.4, respectively, p = 0.41, n = 7–11), respectively) (Fig. S1C and D). Additionally, *Angptl3* gene transfer had no influence on glucose levels in the glucose tolerance test (p ≥ 0.48) (Fig. S1E). Despite the similar Angptl3 levels, the *Angptl3* gene transfer group exhibited increased plasma levels of cholesterol compared with the vector transfer group (total cholesterol: 7.0 ± 2.2 mmol/L *vs*. 8.7 ± 2.1 mmol/L, p = 0.036, n = 12–15; total triglyceride: 1.4 ± 0.5 mmol/L *vs*. 1.3 ± 0.6 mmol/L, p = 0.73, n = 6–10) (Fig. S1F).

By H&E analysis, aortic plaque size was much higher in the *Angptl3* gene transfer group than in the vector group (26.5 ± 4.8 × 10^4^ μm^2^ *vs*. 33.1 ± 8.8 × 10^4^ μm^2^, respectively, p = 0.017, n = 17–20) (Fig. S1G). By immunohistochemistry staining, the numbers of CD68^+^ and α-SMA^+^ cells in the lesion in *Angptl3* transfer mice were both significantly increased compared with the pooled control group (CD68^+^ macrophages: 146.9 ± 18.0 cells/plaque *vs*. 165.6 ± 20.3 cells/plaque, p = 0.031, n = 11–13; α-SMA^+^ smooth muscle cells: 146.8 ± 12.2 cells/plaque *vs*. 172.8 ± 12.5 cells/plaque, p = 0.0001, n = 9–15) (Fig. S1H and I). Representative images of H&E, CD68, and α-SMA staining are shown in Fig. S1J–L.

### Hepatic *Angptl3* overexpression increased Angptl3 levels and plaque progression in *ApoE*^-/-^ mice

As described above, AAV-mediated transfer of *Angptl3* cDNA did not significantly elevate Angptl3 protein expression in HFD-fed *Ldlr*^-/-^ mice. This may be attributable to HFD-mediated induction of relatively high levels of Angptl3. Moreover, plasma levels of triglyceride levels were similar between two groups, indicating that the effects of HFD might partially override gene transfer on ANGPTL3 induction. To exclude the impact of HFD on atherosclerosis, the same gene transfer experiments were repeated in *ApoE*^-/-^ mice on a chow diet.

**Fig. 1:**
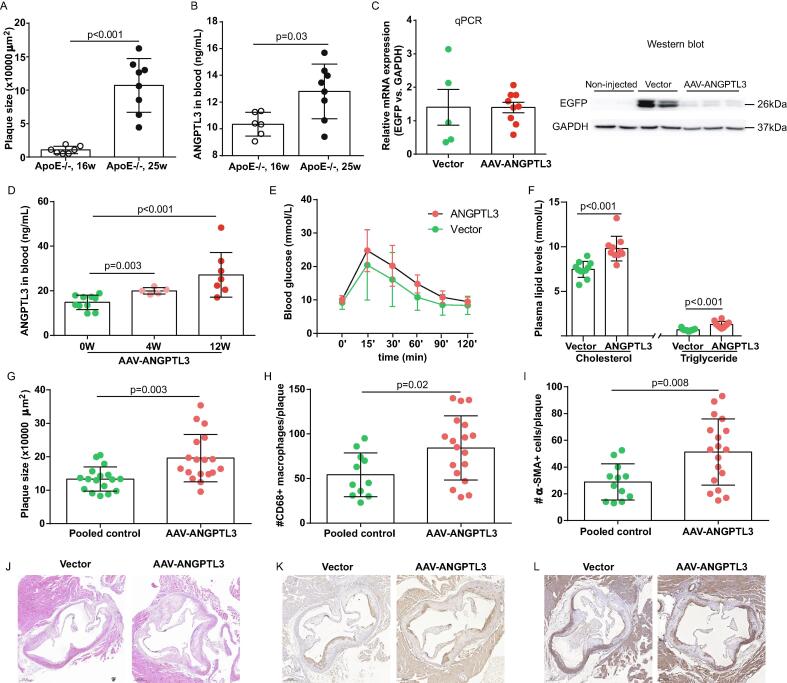
**Effects of hepatic overexpression of *Angptl3* on cholesterol levels and plaque progression in hypercholesterolaemia *Ldlr^-^*^/-^ mice. A,** Plaque size in *ApoE*^-/-^ mice at 16 and 25 weeks of age. n = 7–8. **B,** ELISA of circulating Angptl3 levels in *ApoE*^-/-^ mice at 16 and 25 weeks of age. n = 6–8. **C,** qPCR and western blot analysis of EGFP expression in the liver after 12 weeks of AAV-mediated gene transfer. n = 5–9. **D,** ELISA quantification of circulating Angptl3 levels following *Angptl3* gene transfer. n = 5–10. **E,** Glucose tolerance test. n = 5–6. **F,** Total cholesterol and triglyceride levels in the plasma. n = 10–11. **G,** Plaque size in the aorta. n = 17–18. **H and I,** Numbers of CD68^+^ macrophages and α-SMA^+^ smooth muscle cells in plaque. n = 11–18. **J,** Representative H&E images, Scale bar: 200 μm; **K and L,** Representative of CD68^+^ and α-SMA^+^ staining of cells in plaque, Scale Bar: 200 μm. Un-paired T test was used to compare plaque size, number of CD68^+^ cells and α-SMA^+^ cells (A, G–I). Mann-Whitney test was used to compare blood ANGPTL3 levels (B and D), glucose levels (E), and total cholesterol and triglyceride after gene transfer (F).

The plaque size was increased with age, from 10,713 μm^2^ in 16-week-old *ApoE*^-/-^ mice to 107,070 μm^2^ in 25-week-old *ApoE*^-/-^ mice, as did plasma Angptl3 levels, which showed a 1.2-fold increase with age (p < 0.05 for both, n = 6–8) (Fig. 1A and B). Twelve weeks after gene transfer, EGFP expression was detected in the liver, as shown by qPCR and western blotting (Fig. 1C). Quantified by ELISA, AAV-mediated gene transfer induced 1.3-fold and 1.8-fold increases in circulating Angptl3 levels at weeks 4 and 12 after gene transfer, respectively, compared with non-injected controls (baseline: 14.8 ± 3.3 ng/mL; week 4: 19.9 ± 1.4 ng/mL; week 12: 27.1 ± 10.0 ng/mL; p < 0.01 for both, n = 5–10) (Fig. 1D). Hepatic overexpression of *Angptl3* in *ApoE*^-/-^ mice not only impaired glucose tolerance (Area Under Curve: 64.8 ± 20.5 *vs.* 80.5 ± 24.8, p < 0.007, n = 5–6) (Fig. 1E), but also elevated plasma levels of cholesterol and triglyceride, compared with the vector group (cholesterol: 7.46 ± 0.89 mmol/L *vs*. 9.80 ± 1.39 mmol/L; triglyceride: 0.69 ± 0.14 mmol/L *vs*. 1.28 ± 0.36 mmol/L; p < 0.0001 for both; n = 10–11) (Fig. 1F).

Similar to *Ldlr*^-/-^ mice, aortic plaque size was comparable between the non-injected and vector-injected control groups of *ApoE*^-/-^ mice (p = 0.65). Thus, these two groups were pooled. H&E analysis revealed that AAV-mediated *Angptl3* transfer resulted in a 1.5-fold increase in aortic plaque size compared with pooled controls (13.4 ± 3.7 × 10^4^ μm^2^ *vs.* 19.6 ± 7.0 × 10^4^ μm^2^, p = 0.003, n = 17–18) (Fig. 1G). By immunostaining, the numbers of CD68^+^ macrophages and α-SMA^+^ smooth muscle cells in plaque were 1.6- and 1.8-fold higher, respectively, in mice injected with AAV-ANGPTL3 than controls (CD68^+^ macrophages: 54.2 ± 24.6 *vs*. 84.3 ± 36.0, p = 0.02, n = 12–18; α-SMA^+^ smooth muscle cells: 28.9 ± 13.5 *vs*. 51.3 ± 24.7, p = 0.008, n = 11–18) (Fig. 1H and I). Representative images of H&E, CD68, and α-SMA staining are shown in Fig. 1J–L.

### *Angptl3* deficiency attenuated plaque progression in *ApoE*^-/-^ mice

**Fig. 2:**
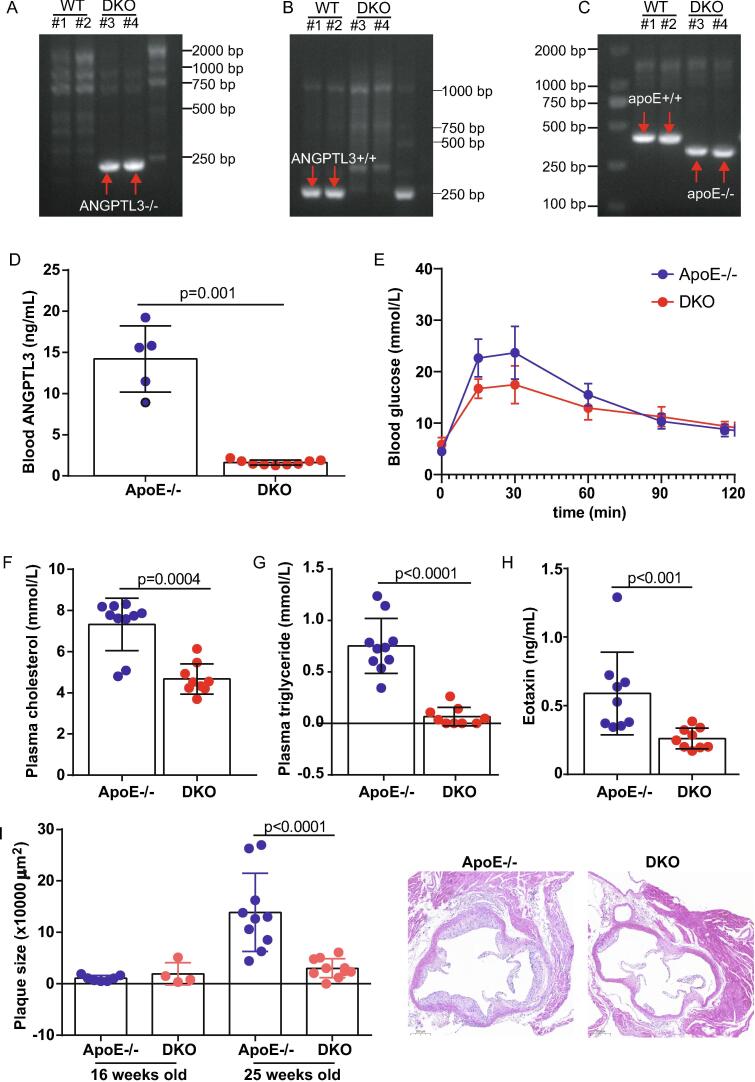
**Attenuation of plaque progression in *ApoE*^-/-^ mice with *Angptl3* deficiency.** Deletion of *Angptl3* exons 1–7 using spCas91.1 technology, followed by crossing of *Angptl3*^-/-^ and *ApoE*^-/-^ mice to generate DKO mice. **A–C,** PCR products amplified by primers against *Angptl3*^-/-^, *Angptl3*^+/+^, and *ApoE*^-/-^. **D,** ELISA quantification of plasma levels of Angptl3 in *ApoE*^-/-^ and DKO mice. n = 5–9. **E,** Glucose tolerance test. n = 4–11. **F,** Fasting plasma levels of cholesterol and triglyceride. n = 9–10. **G,** Lipoprotein lipase (LPL) activity. n = 6–12. **H,** Plasma levels of eotaxin. n = 9 (I) H&E analysis of plaque. Scale Bar: 200 μm. n = 4–10.

To further explore the pathological effects of Angptl3, *Angptl3*^-/-^ mice were created using Cas9 technology. *Angptl3*^-/-^ and *ApoE*^-/-^ mice were crossed to establish double knockout (DKO) mice (Fig. 2A–C). General characterization of *ApoE*^-/-^ and DKO mice was listed in Table S1. ELISA quantification of circulating Angptl3 levels in mice at 25 weeks of age showed a dramatic reduction in DKO mice compared with age-matched *ApoE*^-/-^ littermates (14.2 ± 4.0 ng/mL *vs*. 1.6 ± 0.3 ng/mL, p = 0.001, n = 5–9) (Fig. 2D). When challenged by 20 % glucose, glucose levels were lower in DKO mice than in *ApoE*^-/-^ mice (area under the curve: 1810.0 ± 111.6 *vs*. 1551.0 ± 93.1, p = 0.0006, n = 4–11) (Fig. 2E). Compared with *ApoE*^-/-^ littermates, fasting plasma levels of cholesterol and triglyceride were reduced by 36 % and 91 %, respectively, in DKO mice (cholesterol: 7.3 ± 1.3 mmol/L *vs*. 4.7 ± 0.7 mmol/L; triglyceride: 0.75 ± 0.27 mmol/L *vs*. 0.07 ± 0.09 mmol/L, p ≥ 0.0004 for both, n = 9–10) (Fig. 2F and G).

Next, the circulating cytokine and chemokine profile was established. Quantified by MILLIPLEX® MAP, plasma levels of eotaxin levels in DKO mice were decreased by 56 % compared with *ApoE*^-/-^ mice (589.0 ± 301.7 ng/mL *vs.* 261.2 ± 76.1 ng/mL, p = 0.0005, n = 9) (Fig. 2H). The cytokine and chemokine profiles of *ApoE*^-/-^ and DKO mice are summarised in Table S2. H&E analysis showed that plaque sizes were similar in the initial stage of atherosclerosis, but were 79 % smaller in DKO mice than in *ApoE*^-/-^ mice following atherosclerotic progression (13.9 ± 7.6 × 10^4^ μm^2^ *vs.* 3.0 ± 1.9 × 10^4^ μm^2^, p < 0.0001, n = 4–10) (Fig. 2I).

### Angptl3 homing to atherosclerotic plaque

**Fig. 3:**
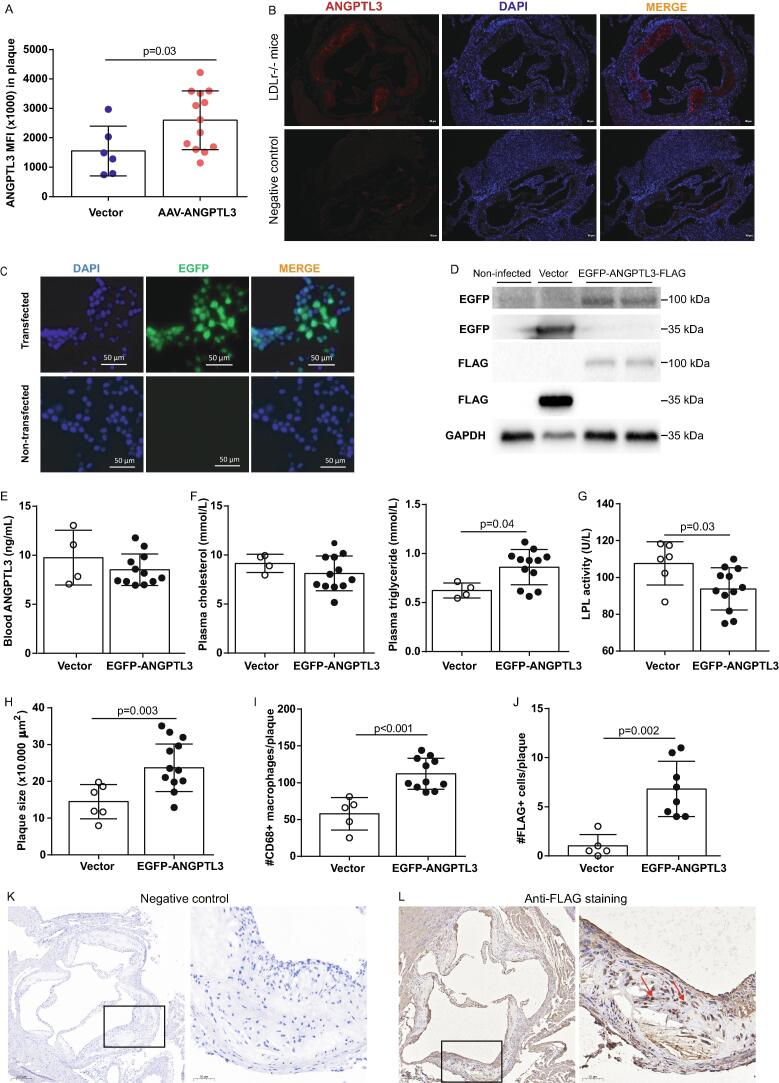
**Angptl3 homing to atherosclerotic plaque. A,** Immunostaining showing the signal density of Angptl3 in plaque from *Ldlr*^-/-^ mice on HFD. n = 6–13. **B,** Representative images of recombinant EGFP-ANGPTL3-FLAG fusion protein. Scale Bar: 80 μm. n = 6–13. **C,** EGFP signal in 293 T cells after vector or AAV-mediated EGFP-ANGPTL3-FLAG cDNA transfer. Scale Bar: 50 μm. n = 3. **D,** Western blotting of EGFP and FLAG expression in the recombinant fusion protein in 293 T cells. n = 4. **E,** ELISA quantification of circulating Angptl3 levels. n = 4–12. **F and G,** Total cholesterol and triglyceride levels in the plasma. n = 4–13. **H,** Plaque size in the aorta. n = 6–12. **I and J,** Numbers of CD68^+^ macrophages and FLAG^+^ cells in plaque. n = 5–11. **K and L,** Representative images of FLAG^+^ cells and negative control cells. Cells marked by black boxes are shown at higher magnification on the right. Cells in the black boxes were further shown on the right. Scale Bar: 50 μm (left) or 200 μm (right). Mann-Whitney test was used in Fig. 3A–J.

To test whether Angptl3 in the circulation could home to plaque, mouse heart sections were immune-stained with anti-mouse Angptl3 antibody. The Angptl3 signal intensity was 1.9-fold higher in plaque from HFD-fed *Ldlr*^-/-^ mice following *Angptl3* gene transfer, compared with the pooled control group (p = 0.03) (Fig. 3A). Representative images of Angptl3 staining in plaque are shown in Fig. 3B.

To further evaluate the homing of Angptl3 to the lesion area in plaque, FLAG-tagged murine Angptl3 was generated by integrating the FLAG peptide sequence into the cDNA construct used for cloning *Angptl3*. Within the same construct, EGFP was also added as a reporter. Two days after transfection of human 293 T cells, EGFP expression could be detected under fluorescent microscopy *in vitro* (Fig. 3C). Western blotting analysis confirmed production of the recombinant EGFP-ANGPTL3-FLAG fusion protein in these cells (Fig. 3D). This plasmid construct was then cloned into the AAV construct and injected into mice via tail vein. Twelve weeks after gene transfer, plasma levels of Angptl3 protein and cholesterol levels were comparable between the vector and AAV-mediated EGFP-ANGPTL3-FLAG cDNA transfer groups (p ≥ 0.25 for both, Fig. 3E and F). However, plasma triglyceride levels were 1.4-fold higher in the EGFP-ANGPTL3-FLAG group than in the vector group (0.62 ± 0.08 mmol/L *vs*. 0.86 ± 0.18 mmol/L, p = 0.04, n = 4–12 per group) (Fig. 3F). To testify the effect of LPL activity by ANGPTL3 gene transfer, plasma samples of vector and ANGPTL3 gene transferred groups were subjected for LPL activity measurement. By ELISA, LPL activity was 13 % lower in EGFP-ANGPTL3-FLAG cDNA transfer group than that of vector group (107.7 ± 11.8 U/L *vs*. 93.7 ± 11.5, n = 6–12, p = 0.03) (Fig. 3G). These data indicate that despite ANGPTL3 levels were similar between vector and ANGPTL3 gene transferred groups, the activity of ANGPTL3 was elevated as evidenced by the inhibition of LPL activity.

Accordingly, plaque sizes and the numbers of CD68^+^ macrophages in plaque were 1.6- and 1.9-fold higher, respectively, in mice with *Angptl3* overexpression (p < 0.01 for both) (Fig. 3H and I). Under immunostaining, FLAG^+^ cells were barely detectable in the vector group, but were prominent the EGFP-ANGPTL3-FLAG group (1.0 ± 1.2 *vs*. 6.8 ± 2.8 cells per plaque, p = 0.002, n = 5–8) (Fig. 3J). Representative images of FLAG staining are shown in Fig. 3K–L.

### Co-localisation of Angptl3 and macrophages in plaque

**Fig. 4:**
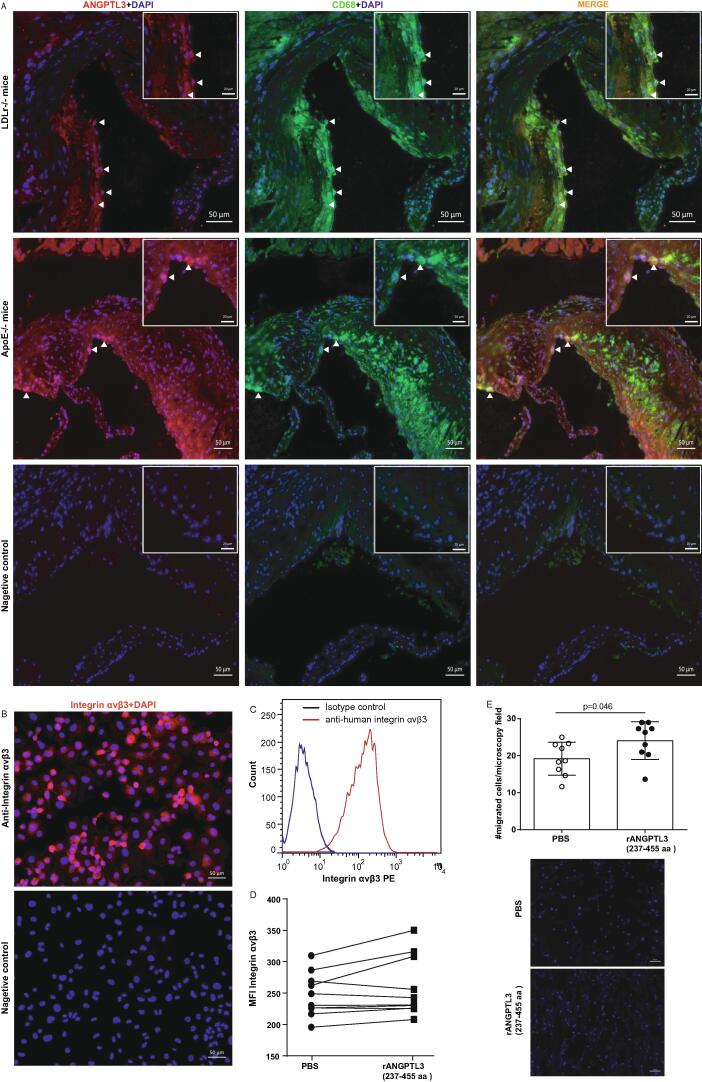
**Co-localisation of Angptl3 and macrophages in plaque. A,** Immunostaining of heart sections from *Angptl3*-transferred HFD-fed *Ldlr*^-/-^ mice and chow diet-fed *ApoE*^-/-^ mice probed with antibodies against Angptl3 and CD68. Co-localisation of both signals were evaluated under confocal fluorescent microscopy. Scale Bar: 50 μm. n = 6–8. **B,** Immunocytochemistry staining of integrin αvβ3 expression in THP-1 cells. Scale Bar: 50 μm. **C,** FACS plot of integrin αvβ3 expression in THP-1 cells. n = 6. **D,** MFI of integrin αvβ3 in THP-1 cells treated with or without ANGPTL3 FBN domain. n = 10. Paired T-test was used to compare MFI before and after ANGPTL3 stimulation. E, THP-1 cells (5 × 10^4^) were subjected onto migration assay. PBS or 50 μg/ml ANGPTL3 was added in the lower chamber. After 6 h of migration, migrated cells were numerated under fluorescent microscopy. Scale bar: 100 μm.

After observing that CD68^+^ macrophages and Angptl3^+^ cells displayed a common pattern of distribution, mouse heart sections from HFD-fed *Ldlr*^-/-^ mice and chow-fed *ApoE*^-/-^ mice were probed with anti-mouse CD68 and anti-mouse Angptl3 antibodies (Abs). Confocal fluorescent microscopy revealed co-localisation of signals for both Abs within the same cells in both genotypes of *Angptl3*-transfer mice (Fig. 4A).

To test whether Angptl3 could act through macrophages, human THP-1 cells were stained for an antibody against integrin αvβ3, the receptor for Angptl3. Immunocytochemistry revealed abundant expression of integrin αvβ3 in THP-1 cells under confocal fluorescent microscopy (Fig. 4B). Fluorescence-activated cell sorting (FACS) analysis confirmed the expression of integrin αvβ3 in THP-1 cells (Fig. 4C). Compared with PBS-treated control, exposure to recombinant Angptl3 FBN-domain modified neither the proportion of integrin αvβ3^+^ cells nor the mean fluorescent intensity (MFI) of this receptor on THP-1 cells (% integrin αvβ3 + cells: 97.9 ± 0.5 % *vs.* 98.1 ± 0.6, p = 0.79, n = 3 for each; integrin αvβ3^+^ MFI: 247.3 ± 34.8 *vs*. 259.4 ± 48.0, p = 0.09, n = 10 for each) (Fig. 4D). To testify whether integrin αvβ3 was functional, THP-1 cells were subjected for migration assay where PBS or Angptl3 was added in the lower chamber. After 6 h of migration, the number of migrated cells was 1.3-fold higher toward Angptl3 than control (#migrated cells per microscopy field: 19.2 ± 4.5 *vs.* 24.1 ± 5.1, n = 9, p = 0.046) (Fig. 4E). These data imply that the regulation of Angptl3 on macrophage function could be mediated by signal pathways downstream of Angptl3/ integrin αvβ3.

### Phosphorylation profile in THP-1 cells exposed to ANGPTL3

**Fig. 5:**
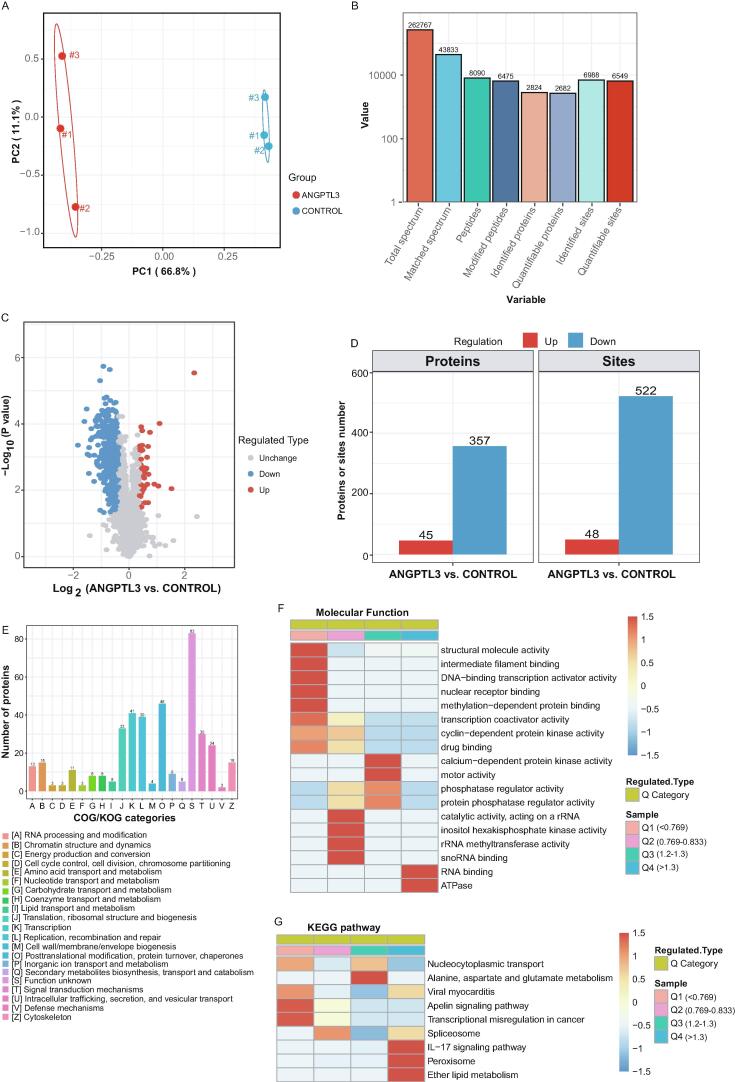
**Protein phosphorylation profiles in THP-1 cells treated with ANGPTL3.** Phosphor proteomics LC-MS analysis to explore signal transduction downstream of ANGPTL3 in THP-1 macrophages stimulated with ANGPTL3 FBN domain or PBS control. **A,** PCA plot. **B,** Summary of secondary total spectrum identified by phospho-proteomics. **C,** Volcano plot of differentially expressed phosphorylated proteins. **D,** Numbers of proteins and sites. **E,** Numbers of proteins classified using the COG database. **F and G,** Enrichment analyses of molecular functions and KEGG pathways categorised by upregulated or downregulated protein phosphorylation site.

Previously, the ANGPTL3 FBN domain was shown to regulate cell function by promoting phosphorylation pathways in endothelial cells or adipocytes [11], [12]. To elucidate the mechanisms underlying the ANGPTL3-mediated modulation of macrophages, THP-1 cells were serum-deprived overnight and then stimulated with PBS or ANGPTL3 FBN domain for 15 min. Because phosphorylated proteins are highly attributed to the protein expression, the dysregulated phosphorylated proteins were normalized by protein levels for precise analysis. Therefore, cells were digested for proteomics and phospho-proteomics mass spectrometry. The identified peptide length distribution and peptide mass distribution showed good quality control in both proteomics and phospho-proteomics (Fig. S2A–D). Using principal component analysis (PCA), the individual plot showed a separation between the two groups in both phospho-proteomics and proteomics (Fig. 5A and Fig. S2E). Number of peptides per protein distribution in proteomics was presented in Fig. S2F.

For phospho-proteomics, totalled 262, 767 spectra were obtained, of which 43,833 spectra were matched to the existing database. Of the 43,833 matched spectra, 6,475 phosphorylation sites on 2,824 proteins were identified (Fig. 5B). A volcano plot was used to illustrate the differentially expressed proteins at ANGPTL3/control ratios of ≥ 1.3 or ≤ 1/1.3 (Fig. 5C). In total, 45 proteins and 48 phosphorylation sites were upregulated by ANGPTL3 in THP-1 cells (Fig. 5C and D). After classification using the cluster of orthologous groups of proteins (COG) database, 83 proteins with unknown function were obtained. Except that, the top three functional categories of these proteins involved post-translational modification, transcription, and replication (Fig. 5E).

The molecular function and Kyoto Encyclopaedia of Genes and Genomes (KEGG) pathways were analysed on the basis of upregulation or downregulation of phosphorylation sites (Fig. 5F and G). Proteins with ANGPTL3-induced phosphorylation participated in the IL-17 signalling pathway, peroxisomes, and ether lipid metabolism (Fig. 5G).

### ANGPTL3 FBN domain reinforced inflammatory cytokine production in THP-1 cells

**Fig. 6:**
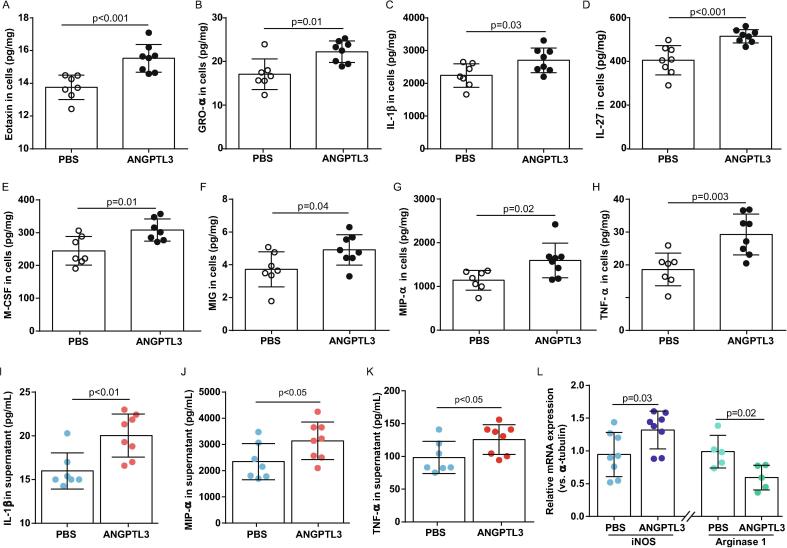
**ANGPTL3 FBN domain reinforced inflammatory cytokine production in THP-1 cells.** Cytokines, chemokines, and growth factor measurements in THP-1 cell extracts and culture supernatants treated with or without ANGPTL3 FBN domain for 24 h: levels of the indicated cytokines in cell extracts **(A–H)** and supernatants **(I–K)** n = 7–8. **L,** THP-1 cells were treated with PBS or ANGPTL3 FBN domain for 24 h. qPCR was performed to study iNOS and arginase 1 expression.

To validate the phospho-proteomic findings, the inflammatory profiles of THP-1 cells treated with ANGPTL3 FBN domain were obtained. After a 24-h exposure to PBS or ANGPTL3 FBN domain, a panel of cytokines, chemokines, and growth factors in the cellular content and supernatant of THP-1 cell cultures were determined using the MILLIPLEX MAP assay. After normalisation for the amount of protein loaded, treatment with ANGPTL3 FBN domain was found to elicit production of a series of inflammatory cytokines, including eotaxin, GRO-α, IL-1β, IL-27, M−CSF, MIG, MIP-α, and TNF-α (Fig. 6A–H). The concentrations of IL-1β, MIP-α, and TNF-α in the supernatant were also increased by 1.3–1.4-fold in ANGPTL3 FBN domain-treated cells compared to those in control cells (Fig. 6I–K). Tables S3 and S4 summarise the inflammatory profiles in THP-1 cells and culture supernatants.

As shown in the inflammatory cytokine/chemokine profiles, hallmarks of M1 macrophages were elevated such as IL-1β [13], [14] and TNF-α [13], [14] in ANGPTL3-treated THP-1 cells. Given that iNOS [13], [14] and arginase 1 [13], [14] are markers of M1 and M2 macrophages, respectively, we further testified the potential of ANGPTL3 in phenotype switch. After 24 h of treatment, cells were harvested for RNA extraction. qPCR data revealed that the relative iNOS mRNA expression was 0.95 at baseline but increased to 1.32 in ANGPTL3-stimulated cells (0.95 ± 0.34 *vs*. 1.32 ± 0.29, n = 8, p = 0.03). Conversely, mRNA expression of arginase 1 was reduced 40 % by ANGPTL3 compared with control (0.99 ± 0.25 *vs*. 0.59 ± 0.19, n = 5, p = 0.02) (Fig. 6L).

Taken together, these data suggest that ANGPTL3 FBN domain was potent to regulate phenotype switch toward M1 macrophages.

### ANGPTL3 FBN domain promoted TLR4 expression via Akt phosphorylation

**Fig. 7:**
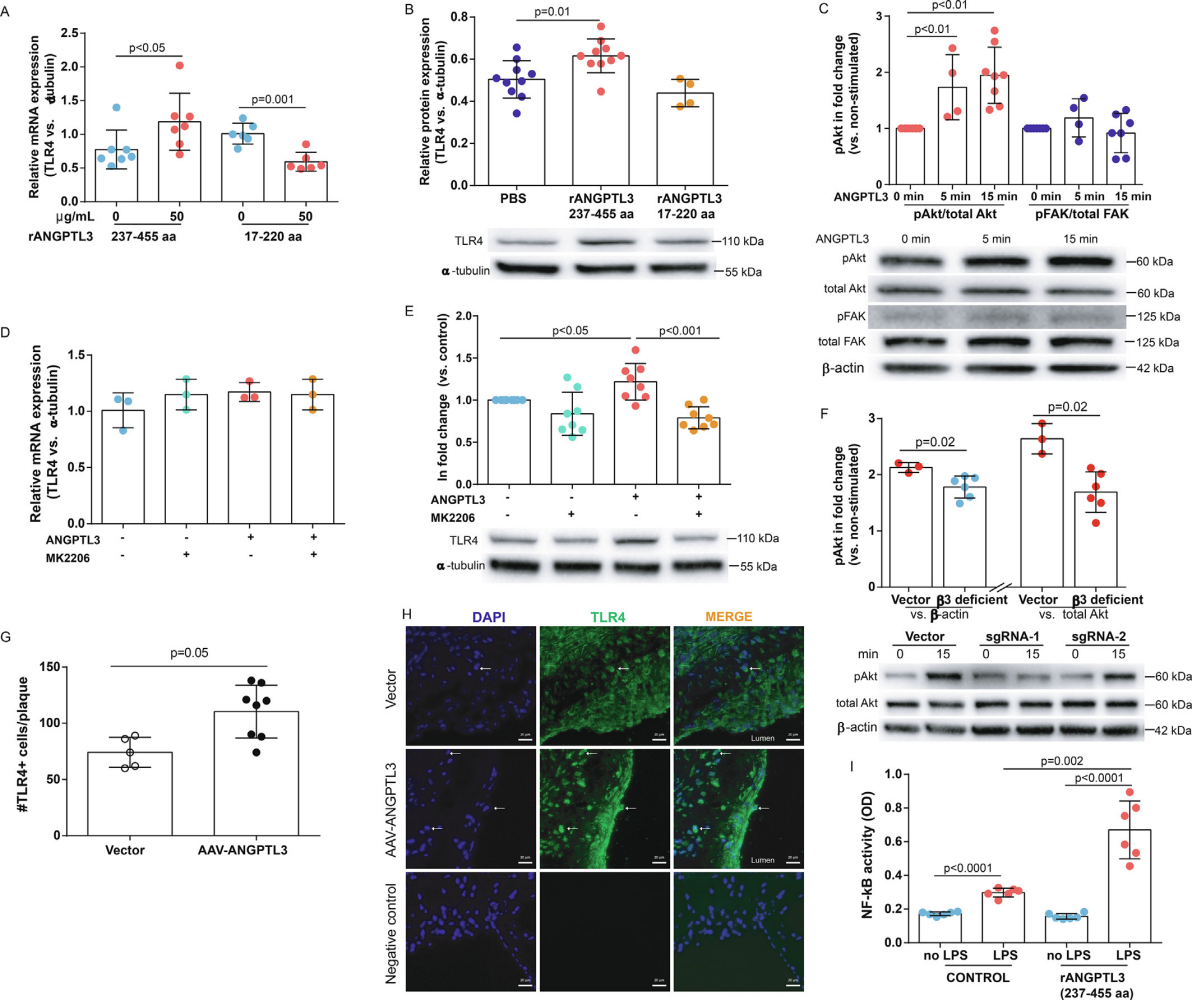
**ANGPTL3 FBN domain promoted TLR4 expression via Akt phosphorylation**. **A and B**, THP-1 cells were treated with or without ANGPTL3 FBN domain for 24 h. mRNA and protein expression of TLR4 were analysed by qPCR and western blot, respectively. n = 4–10. **C**, After overnight fasting, THP-1 cells were simulated with PBS, 50 μg/ml ANGPTL3 FBN domain for 5 or 15 min. Western blot was performed to assess Akt and FAK phosphorylation. n = 4–7. **D and E**, THP-1 cells were exposed to ANGPTL3 FBN domain in the presence or absence of MK2206 for 24 h. Western blot and qPCR were performed to study TLR4 expression. n = 8 for western blot. **F,** THP-1 cells were infected with sgRNAs targeting RGD sequence of integrin β3 using lentivirus-Cas9 system. After 3 days and infection followed by 7 days of selection, cells were stimulated with 50 μg/ml Angptl3 for 0 or 15 min. Cells were harvested for western blot analysis of pAkt, total Akt and β-actin. **G,** Heart sections were probed with anti-mouse TLR4 antibody and the number of TLR4 + cells in plaque was analysed. **H,** Representative images of TLR4 + staining sections were presented. White arrows indicated TLR4 + cells in the plaque. Scale bar: 20 μm. **I,** THP-1 cells were pre-treated with FBN domain ANGPTL3 for 24 h and then exposure to 100 ng/ml LPS for 15 min. Nuclear proteins were extracted to determine NF-κB activity. n = 6.

TLR4-mediated IL-17 production in macrophages has been reported in diseases such like arthritis and hepatitis [15], [16]. Likewise, the inflammatory profiles also indicated the activation of TLR4/NF-κB pathways. To testify that, THP-1 cells were treated with FBN-domain of ANGPTL3 (0 or 50 μg/mL) for 24 h. Examination of TLR4 expression by qPCR and western blotting showed that stimulation with ANGPTL3 FBN domain increased mRNA and protein expression of TLR4 by 1.7-fold and 1.3-fold, respectively, compared with controls (qPCR: 0.77 ± 0.29 *vs.* 1.19 ± 0.42, p = 0.038, n = 6–7; western blotting: 0.51 ± 0.03 *vs.* 0.64 ± 0.09, p = 0.009, n = 4–10) (Fig. 7A and B). Despite a 42 % decrease in mRNA levels overall in THP-1 cells treated with N-terminal ANGPTL3, TLR4 protein expression was similar between PBS-treated controls (qPCR: p = 0.001; western blotting: p = 0.42) (Fig. 7A and B).

We further investigated whether the regulation of TLR4 expression by ANGPTL3 was mediated via phosphorylation. THP-1 cells were stimulated with PBS or ANGPTL3 FBN domain for 15 min. Using western blot analysis, exposure to ANGPTL3 FBN domain elicited 1.8-fold activation of Akt compared with un-treated controls (p < 0.05 for both) (Fig. 7C). By contrast, FBN domain stimulation did not induce FAK phosphorylation compared to control (p = 0.69) (Fig. 7C). Similar results were obtained when anti-β-actin was used as the internal control for pAkt (baseline: 0.33 ± 0.05 for baseline, 0.48 ± 0.08 for 5 min, and 0.58 ± 0.03 for 15 min, n = 4 for each, p < 0.05 for both). When normalised by β-actin, pFAK levels remained unaltered before and after ANGPTL3 stimulation (p ≥ 0.11 for both).

To verify whether pAkt was involved in ANGPTL-3-mediated TLR4 expression, THP-1 cells were treated with ANGPTL3 FBN domain with or without pAkt inhibitor for 24 h. Using western blot analysis, inhibition of Akt phosphorylation attenuated TLR4 expression induced by ANGPTL3 (p = 0.0002 for ANGPTL3 *vs.* ANGPTL3 + MK2206) (Fig. 7D). Nevertheless, the addition of MK2206 did not modify TLR4 mRNA levels (p = 0.90 for ANGPTL3 *vs.* ANGPTL3 + MK2206) (Fig. 7E). To explore whether ANGPTL3 activated pAkt via binding to RGD sequence in integrin β3 [17], [18], two single guide RNAs were designed to target RGD sequence using CRISPR/Cas 9 system. They were packed into lentivirus for infecting THP-1 cells. Three days after infection, cells were selected with puromycin and blasticidin S for 7 days. Compared with vector group, sgRNAs did not alter integrin β3 expression by Western blot analysis (p ≥ 0.80). Upon ANGPTL3 stimulation, total Akt levels were unchanged among all groups (p ≥ 0.45). However, the ratio of pAkt/total Akt was reduced 36 % and 37 %, respectively, in β3 deficient cells compared with vector group (Vector: 2.6 ± 0.3; sgRNAs-1: 1.7 ± 0.3; sgRNAs-2: 1.7 ± 0.5). Because both sgRNAs resulted in similar pAkt induction (p = 0.9), the data of pAkt/total Akt were pooled into one group. By Mann-Whitney analysis, Akt phosphorylation was significantly reduced in integrin β3 deficient cells when compared to vector group (pAkt/total Akt: 2.6 ± 0.3 for vector; 1.7 ± 0.4 for integrin β3 deficient group, n = 3–6, p = 0.02) (Fig. 7F). Similar findings were observed when normalised by β-actin (pAkt/β-actin: 2.1 ± 0.1 *vs.* 1.8 ± 0.2, n = 3–6, p = 0.02) (Fig. 7F). These data hint that Angptl3 promoted Akt activation via binding to RGD of integrin β3.

After we observed increased TLR4 expression in ANGPTL3-treated THP-1 cells, heart sections of gene transferred *ApoE*^-/-^ mice were probed with anti-TLR4. The number of TLR4^+^ cells was 74.2 per plaque in vector group but increased to 110.4 per plaque in ANGPTL3 group (#TLR4^+^ cells per plaque: 74.2 ± 13.3 *vs*. 110.4 ± 23.6, n = 5–8, p = 0.01) (Fig. 7F and H).

### Increased NF-kB activity via ANGPTL3 in THP-1 cells

To explore whether TLR4 was functional, THP-1 cells were first incubated with PBS or ANGPTL3 for 24 h and then stimulated with 100 ng/mL LPS for 15 min. Without LPS stimulation, NF-κB activity was comparable between PBS and ANGPTL3-treated cells (p = 0.10) (Fig. 7I). Upon LPS treatment, NF-kB activity was 1.8-fold increase in PBS-treated THP-1 cells, which climbed to a 4.2-fold increase when the cells were pre-treated with ANGPTL3 FBN domain (p = 0.002, Fig. 7I).

### Detection of ANGPTL3 signals in human plaque

**Fig. 8:**
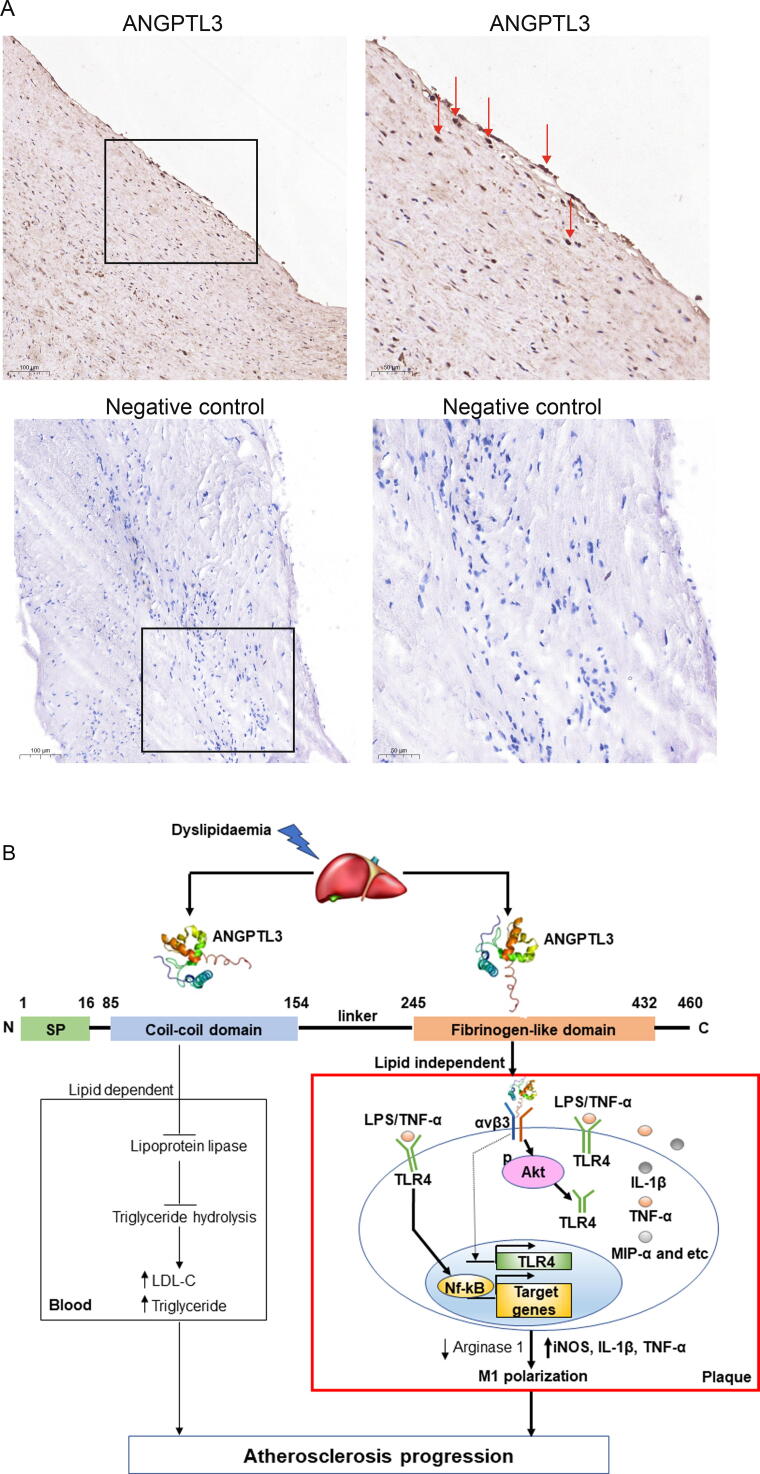
**ANGPTL3 in human plaque. A,** Immunostaining of ANGPTL3 signals inside paraffin sections of human plaque specimens. Negative controls were performed by the omission of primary antibody against ANGPTL3. N = 5. Scale Bar: 500 μm (left) or 1000 μm (right). **B,** a proposed model. Under stimuli such as high-fat diet, increased ANGPTL3 protein is produced in liver and secreted into blood. The coil-coil domain of Angptl3 inhibits lipoprotein lipase activity, resulting in elevated LDL-c and triglyceride levels in the blood. The Fibrinogen domain of Angptl3 homes to plaque where it binds to integrin αvβ3 in macrophages. Upon binding, Angptl3 promoted Akt phosphorylation and enhanced TLR4 production and NF-κB activity, leading to increased inflammatory cytokines and chemokines in the blood. Ultimately, Angptl3 accelerates atherosclerotic progression.

Finally, we investigated the presence of ANGPTL3 in atherosclerotic plaque dissected from the cerebrovascular artery of five patients under the guidance of angiography. Immunostaining of paraffin-embedded sections confirmed the presence of ANGPTL3 signals in these plaque specimens (Fig. 8A).

## Discussion

The main findings of the study are summarised as follows. First, Angptl3 held pro-atherogenic properties, as evidenced by increased plaque size in HFD-fed *Ldlr*^-/-^ hypercholesterolaemia mice and chow-fed *ApoE*^-/-^ mice under hepatic overexpression of *Angptl3*. Compared with *ApoE*^-/-^ littermates, DKO mice had lower cholesterol and triglyceride levels and smaller plaque size. Second, besides modulation of cholesterol and triglyceride levels in the blood, Angptl3 exhibited homing to plaque, where it directly regulated macrophage function. Specifically, via Akt phosphorylation, ANGPTL3 promoted TLR4 expression, triggered pronounced NF-kB activation in response to LPS, and elicited inflammatory cytokine production and M1 polarization in THP-1 macrophages. *In vivo*, TLR4 expression was increased to facilitate plaque inflammation and progression in ANGPTL3 gene transferred mice. The proposed model is summarized in Fig. 8B.

Dyslipidaemia and chronic inflammation orchestrate the pathogenesis of atherosclerosis. In a *meta*-analysis involving 23 studies, plaque volume was assessed by intravascular ultrasound in 7,407 patients receiving lipid-lowering therapies for 11 to 104 weeks. After adjusting for cofounding factors, 1 % regression of atherosclerotic plaque was correlated with a 25 % decrease in the odds of MACE [19]. In line with that, data from mouse models and *in vitro* studies have demonstrated that hypercholesterolaemia not only promotes monocyte and neutrophil activation, but also stimulates haematopoietic stem/progenitor cell proliferation and differentiation toward increased inflammatory cell production, both of which reinforce plaque progression [20]. Therefore, reduction of LDL-c does not always increase cardiovascular protection [3]. Likewise, anti-inflammatory strategies only partially circumvent the occurrence of MACE [4], [5]. These phenomena indicate that control of either hyperdyslipidaemia or inflammation is not sufficient to suppress atherosclerotic plaque progression.

In the initial stage of atherosclerosis, subendothelial penetration of modified LDL triggers endothelial cell dysfunction, attracting the homing of circulating inflammatory cells to the inflamed endothelium, and aggravating inflammation and plaque formation [20]. Macrophages with excessive uptake of lipoprotein are transformed into the lipid-laden foam cells that are the hallmark of atherosclerosis. Nevertheless, not all such macrophages are identical. So far, four phenotypes classified by metabolic state and microenvironment have been identified *in vitro* [21], among which the M1 and M2 phenotypes are the most well studied. In the setting of atherosclerotic cardiovascular disease, activated M1 macrophages trigger and maintain inflammation, whereas activated M2 macrophages suppress inflammation [13]. How to directly inhibit the atherosclerotic properties of macrophages in plaque remains an unmet challenge.

Using FLAG-labelled protein for *in vivo* tracing and double-fluorescent staining, we demonstrated that Angptl3 was recruited into plaque, where it acted directly on macrophages. *In vitro*, exposure of ANGPTL3 FBN domain to cultivated THP-1 cells increased TLR4 expression and NF-kB activation in response to LPS, leading to increased production of inflammatory cytokines, such like IL-1β, TNF-α, and IL-27. These findings indicated the polarisation of the M1 phenotype upon ANGPTL3 stimulation. *In vivo*, circulating eotaxin levels in DKO mice were significantly increased compared with *ApoE*^-/-^ littermates. Eotaxins are C–C motif chemokines that are chemo-attractants for eosinophils, basophils, and T helper 2 lymphocytes [22]. Apart from its involvement in allergic diseases, eotaxin has been associated with neurodegeneration and impaired cognition [23]. In a rat model of heart transplantation, eotaxin mRNA expression was detected in macrophages infiltrating the rejected heart, indicating its role in M1 macrophage polarisation [24]. Our *in vitro* cytokine profiling revealed an increase in eotaxin production in ANGPTL3-treated THP-1 cells. Accordingly, plasma levels of eotaxin were reduced in *ApoE*^-/-^ mice with *Angptl3* deficiency. These data suggested that macrophages may serve as a novel source of eotaxin production. However, the mechanism through which eotaxin participates in atherosclerosis progression needs further investigation.

There are several limitations to this study. First, by phospho-proteomics, 6,988 phosphorylation sites in 2,824 proteins were identified downstream of ANGPTL3/integrin αvβ3 in THP-1 cells. They were located in the cytosol, spliceosome, or nucleoplasm. These proteins were involved in a variety of molecular functions, including histone binding and translation initiation factor binding. However, it was not feasible to validate each phosphorylation site with currently available commercial antibodies. Second, blocking Akt phosphorylation attenuated TLR4 protein expression but not mRNA levels in THP-1 cells treated with ANGPTL3. How ANGPTL3 regulates TLR4 transcription is unclear. Third, we could not exclude the possibility that the reduced plaque size in DKO mice was at least partially attributable to the significantly lower plasma levels of cholesterol and triglyceride in DKO mice compared with those in *ApoE*^-/-^ mice. Nevertheless, the inflammatory profiles induced by ANGPTL3 in THP-1 cells demonstrated that the pro-atherosclerotic effects of ANGPTL3 were independent of changes in lipid levels. And fourth, using lentivirus-mediated Cas9 system, RGD sequence within integrin β3 was partially deleted in THP-1 cells. Despite infected cells were purified by puromycin and blasticidin S for 7 days, due to limited spCAS9 editing efficiency, mixed clones with different levels of integrin β3 were obtained. Not surprisingly, integrin β3 was comparable among all groups using western blot analysis. Integrin β3 belongs to RGD-binding integrin family in which activation is crucial for activation of downstream signal pathways. In line with that, we detected attenuated Akt activation in mixed clones when stimulated with Angptl3, in comparison to controls. This confirmed that Angptl3 induced pAkt via binding to RGD sequence in integrin β3. For perspective, a stable clone with complete deletion of RGD sequence is required to further dissect the mechanism underlying the regulation of Angptl3 on macrophage phenotype switch.

## Conclusion

Our findings demonstrated that, in addition to elevating cholesterol and triglyceride levels in the blood, ANGPTL3 exhibited homing to plaque, where it directly modulated the inflammatory properties of macrophages, leading to atherosclerotic progression. Targeting ANGPTL3 using mAbs or small interfering RNA could provide health benefits via reductions in blood lipid levels and attenuation of M1 macrophage activation in plaque.

## Ethic statement

All experiments involving animals were conducted according to the ethical policies and procedures approved by the ethic committee of Capital Medical University (AEEI-2023-234). The human study protocol was approved by the competent Institutional review Boards of Beijing Youan hospital (No. LL-2023-146-K). All patients provided written informed consent.

## Declaration of competing interest

The authors declare that they have no known competing financial interests or personal relationships that could have appeared to influence the work reported in this paper.
