# Supplementary material for: ANGPTL3 accelerates atherosclerotic progression via direct regulation of M1 macrophage activation in plaque
Source: J Adv Res. 2024 May 11;70:125–38. doi: 10.1016/j.jare.2024.05.011 (PMC11976407; doi:10.1016/j.jare.2024.05.011)
Supplement: Supplementary Data 1 [file mmc1.docx]

# Supplementary materials

ANGPTL3 accelerates atherosclerotic progression via direct regulation of M1 macrophage activation in plaque

Running title: ANGPTL3-mediated M1 macrophage activation

Yuejie Zhang^a,1^, Cen Yan^a,1^ Yuan Dong^a^, Jiwei Zhao^a^, Xuanyi Yang^a^, Yalan Deng^a^, Li Su^b^, Yang Zhang^c^, Fenghui Sun^d^, Yingmei Feng^a,b,*^

^a^Beijing Institute of Hepatology, Beijing Youan hospital, Beijing 100069, China

^b^Department of Science and Technology, Beijing Youan Hospital, Beijing 100069, China

^c^Neuroscience Research Institute, Peking University Center of Medical and Health Analysis, Peking University, Beijing 100191, China

^d^Department of Neurology, Beijing Youan hospital, Beijing 100069, China

Corresponding author: Yingmei Feng, Beijing Institute of Hepatology, Beijing Youan hospital, Beijing 100069, China.

Email: [yingmeif13@sina.com](mailto:yingmeif13@sina.com); [yingmeif13@ccmu.edu.cn](mailto:yingmeif13@ccmu.edu.cn)

1Equal contribution to the study

# Methods and Materials

## Human subjects

Five patients with coronary heart disease were enrolled in the department of Neurology in Beijing Youan hospital from January 1^st^ 2023 till October 30^th^ 2023. The plaque in the cerebrovascular artery was carefully dissected out using bare wire thrombectomy technique under the guidance of angiography.

## Mice and treatment

In total, 182 male, wild-type, *Ldlr*^-/-^, *ApoE*^-/-^, and DKO mice on the C57BL/6J background were used in the study. For the DKO, exons 1–7 (aa 1-455) in *Angptl3* was completely deleted using CRISPR-cas9 technology. After obtaining stable *Angptl3*^-/-^ mice, they were crossed with *ApoE*^-/-^ mice for more than four generations to create a stable DKO strain. Age-matched *ApoE*^-/-^ mice served as controls for the DKO strain. To study the effects of Angptl3 on atherosclerosis progression, *Ldlr*^-/-^ mice (age, 8 weeks) and *ApoE*^-/-^ mice (age, 23–25 weeks) were administered AAV carrying *Angptl3* cDNA or empty vector via tail vein injection (1 × 10^11^ viral genomes per mouse). Immediately after injection, the *Ldlr*^-/-^ mice were placed on a HFD (1.25% cholesterol, 40% fat) for 12 weeks, and the *ApoE*^-/-^ mice were maintained on a chow diet for 12 weeks.

## Ethics statement

All experiments involving animals were conducted according to the ethical policies and procedures approved by the ethic committee of Capital Medical University (Approval no. AEEI-2023-234). The human study protocol was approved by the competent Institutional review Boards of Beijing Youan hospital (Approval no. LL-2023-146-K). All patients provided written informed consent.

## Mice and treatment

In total, 182 male, wild-type, *Ldlr*^-/-^, *ApoE*^-/-^, and DKO mice on the C57BL/6J background were used in the study. For the DKO, exons 1–7 (aa 1-455) in *Angptl3* was completely deleted using CRISPR-cas9 technology. After obtaining stable *Angptl3*^-/-^ mice, they were crossed with *ApoE*^-/-^ mice for more than four generations to create a stable DKO strain. Age-matched *ApoE*^-/-^ mice served as controls for the DKO strain. When sacrifice, mice were anaesthetized by a subcutaneous injection of mixture containing 100 mg/kg ketamine, 50 μg/mL atropine, and 10 mg/kg xylazine.

### Genotyping

To screen for DKO mice, a 0.3–0.5 cm tail segment was collected for biopsy. Samples were mixed with lysis buffer, incubated at 60°C overnight, spun by centrifugation at 13,300 rpm/min, heated a 95°C for 5 min, then re-spun to collect DNA for PCR-based genotyping (Table 1).

Table 1. Primer sequences and PCR products generated for genotyping.

| Gene | Primer name | Primer sequence (5’-3’) | PCR product |
| --- | --- | --- | --- |
| *ANGPTL3* | KO-G7-F | AGTGCCTATTAGACAGCAAGAAAG | KO:345bp |
|  | KO-G7-R | GAGAAACGACACCCTTCACAG |  |
|  | M-KO-G7-WT-FP | GACAGCAAGAAAGATTCCAGTGA | WT:223bp |
|  | M-KO-G7-WT-RP | TGGTTTCTTTAGCGATGCCAG |  |
| *APOE* | M-APOE-FP | ACAGACTCCACAGCCTCCAGAC | KO:335bp  WT:417bp |
|  | M-APOE-RP | TGTTCCTCCCAGCATCCAGAAG |  |

### Adeno-associated virus (AAV)-mediated gene transfer in mice

As previously reported,[1] murine *Angptl3* cDNA was cloned into plasmid pAAV-EF1A-EGFP-P2A-MSC-3Flag (OBiO Technology, Shanghai Corp, Ltd. China), a serotype 8 AAV vector carrying a thyroxine-binding globulin promoter to powerfully induce transgene expression in hepatocytes.

To study the effects of Angptl3 on atherosclerosis progression, *Ldlr*^-/-^ mice (age, 8 weeks) and *ApoE*^-/-^ mice (age, 23–25 weeks) were administered AAV carrying *Angptl3* cDNA or empty vector via tail vein injection (1 × 10^11^ viral genomes per mouse). Immediately after injection, the *Ldlr*^-/-^ mice were placed on a high-fat diet (HFD) (1.25% cholesterol, 40% fat) for 12 weeks, and the *ApoE*^-/-^ mice were maintained on a chow diet for 12 weeks.

### Angptl3 homing experiments in mice

To investigate whether Angplt3 in the circulation could home to plaque, murine *Angptl3* cDNA was cloned into plasmid pAAV-EF1A-EGFP-MSC-3Flag (OBiO Technology), and administered to *ApoE*^-/-^ mice (age, 25 weeks) by tail vein injection (1 × 10^11^ viral genomes per mouse). Twelve weeks after injection, mice were sacrificed to assess Angptl3 homing by immunostaining.

### Glucose tolerance test

After overnight fasting, *Ldlr*^-/-^ and *ApoE*^-/-^ mice were intraperitoneally injected with 20% glucose (10 μL/g body weight). Blood glucose levels were determined before and at 15, 30, 60, 90, and 120 min after injection (Roche, USA).

### Plasma lipid profiles

After overnight fasting, blood samples were collected and centrifuged at 3000 rpm for 10 min. Plasma levels of total cholesterol and triglyceride were determined using kits (Nanjing Jiancheng Corp., Nanjing, China), following the manufacturer’s instructions.

### Enzyme-linked immunosorbent assay (ELISA)

Plasma levels of Angptl3 of mice in the fasting state were quantified using a mouse Angptl3 assay kit (Cat. 27410; IBL, Japan), following the manufacturer’s instructions.

### Protein extraction

Proteins were extracted from THP-1 cells using RIPA buffer (Solarbio, Beijing, China) mixed with proteinase inhibitors (Roches, Shanghai, China). Protein concentrations were quantified using the bicinchoninic acid (BCA) method (Thermo Scientific, USA).

### Cytokine measurement by MILLIPLEX® MAP

For THP-1 cells, proteins were extracted using RIPA buffer (MILLIPLEX® MAP, USA) and the concentrations were quantified using the BCA method. Equal microgram amounts of protein preparations or equal volumes of supernatants were loaded into 96-well plates and mixed with immobilised premixed magnetic beads based on Luminex xMAP^®^ technology. The following panel of cytokines was determined by MILLIPLEX® MAP (Cat. #HCYTA-60K; Merck KGaA, Darmstadt, Germany), following the manufacturer’s instructions: sCD40L, EGF, Eotaxin, FGF-2, FLT-3L, Fractalkine, G-CSF, GM-CSF, GRO-α, IFN-α2, IFN-γ, IL-1α, IL-1β, IL-1RA, IL-2, IL-3, IL-4, IL-5, IL-6, IL-7, IL-8, IL-9, IL-10, IL-12(p40), IL-12(p70), IL-13, IL-15, IL-17A, IL-17E/IL-24, IL-17F, IL-18, IL-22, IL-27, IP-10, MCP-1, MCP-3, M-CSF, MDC, MIG/CXCL9, PDGF-AA, PDGF-AB/BB, RANTES, TGF-α, TNF-α, TNF-β, and VEGF-A.

For the mouse study, equal volumes of fasted plasma samples were loaded into 96-well plates for MILLIPLEX Multiplex assays of the following cytokines using Luminex technology (Cat. # MCYTOMAG-70K; Merck KGaA): Eotaxin, IFN-γ, IL-1α, IL-1β, IL-2, IL-6, IL-10, IL-17, IP-10, MCP-1, MIP-1α, M-CSF, MIP-2, VEGF, and TNF-α.

### Flow cytometry

To determine whether human macrophages express ANGPTL3 receptor, THP-1 cells were probed with phycoerythrin (PE)-conjugated anti-human integrin αvβ3 (Clone LM609; Millipore, USA) at a dilution of 1:200 for 30 min at 4°C. To study macrophage phenotype, THP-1 cells were probed with phycoerythrin (PE)-conjugated anti-human CD86 (Cat. 374206; Biolegend, USA), Allophycocyanin (APC)-conjugated anti-human CD206 (Cat. 321110; Biolegend, USA) and Fluorescein (FITC)-conjugated anti-human CD68 (Cat. 333806; Biolegend, USA) at a dilution of 5 μl/1× 10^6^ for 30 min at 4°C. After washing with phosphate-buffered saline (PBS), 50,000 cells were acquired by a FACS Aria II (BD). In parallel, cells stained with immunoglobulin isotype IgG1 were used as negative controls.

### Cell culture

THP-1, a human monocyte leukaemia cell line, was obtained from Professor Xunde Xian (Peking University, China). The cells were cultured at a density of 5 × 10^5^ cells/mL in RPMI 1640 medium (Gibco, USA) supplemented with 10% foetal bovine serum (FBS; Gibco, USA) and 1% penicillin/streptomycin solution (Gibco, USA) at 37°C in a 5% CO_2_ incubator. Prior to stimulation, THP-1 monocytes were cultured for 24 h in 6-well plates treated with 100 ng/mL phorbol 12-myristate 13-acetate (PMA). The cells were allowed to adhere and differentiate into macrophages, then were expanded in the absence of PMA for another 24 h. After extensive washing, the cells were exposed to 50 μg/mL N-terminal ANGPTL3, 50 μg/mL ANGPTL3 FBN domain (Sino Biological, Peking, China), or PBS for 24 h, then were collected for further experiments.

HEK 293T cells (Cell Bank/Stem Cell Bank, Chinese Academy of Sciences) were cultured in Dulbecco’s modified Eagle’s medium supplemented with 10% FBS and 1% penicillin/streptomycin solution at 37°C in a 5% CO_2_ incubator.

For phospho-proteomics analysis of phosphorylated proteins, 1 × 10^7^ THP-1 cells were serum deprived overnight, stimulated with 0 or 50 μg/mL ANGPTL3 FBN domain for 15 min, then harvested with cold PBS and stored at liquid nitrogen.

For inhibition experiments, THP-1 cells were pre-incubated with 10 μM MK2206 for 2 hours and then treated with 50 μg/ml Angptl3 for 24 hours. Cells were collected to performed western blot. Alternatively, THP-1 cells were exposed to 100 ng/ml LPS to determine NF-kB activity.

### Cell transfection

In Angptl3-tracing experiment, FLAG-tagged murine Angptl3 was generated by integrating the FLAG peptide sequence into *Angptl3* cDNA. Within the same construct, EGFP was added as a reporter. To testify protein expression, the plasmid was transfected into HEK 293T cells. Briefly, HEK 293T cells were seeded at a density of 20% in a 24-well plate. Three days after seeding, the cells were transfected at a multiplicity of infection of 1 × 10^9^ using lipofectamine (Obio Technology,) in accordance with the manufacturer’s instructions. Three days after transfection, EGFP expression was assessed under fluorescent microscopy (Nikon, Japan). Cells were harvested for Western blotting.

The *Integrinβ3* deficient cells were generated by the CRISPR/Cas9 system. Two 20 bp sgRNAs designed to target exon 2 or exon 3 of *Integrinβ3*, both of which were targeted on Arginine-Glycine-Aspartic (RGD)-binding sites at amino acids 61-203 [2, 3]. They were inserted into LentiCRISPRv2 plasmid and then transfected to 293T cells to prepare sgRNA-Cas9 lentivirus (Obio Technology, Shanghai, China). THP-1 cells were infected with the lentivirus at a multiplicity of infection (MOI) of 60 and cultured in complete medium for 72 hours. The *Integrinβ3* deficient cells were selected by 2 µg/ml puromycin and blasticidin S for 7 days. The integrinβ3 expression was assessed by Western blotting.

### RNA extraction and quantitative PCR (qPCR) analysis

Total RNA was extracted from THP-1 cells using TRIzol Reagent (Cat. 15596018; Thermo Scientific), then was reverse transcribed into cDNA for qPCR. qPCR was conducted using AceQ Universal SYBR master mix (Q711-02, Vazyme, Nanjing, China) (Table 2).

Table 2. Primers used to amplify the indicated genes by qPCR

| Genes | Forward | Reverse |
| --- | --- | --- |
| *TLR4* | TCCTGCGTGAGACCAGAAAG | AATGGAATCGGGGTGAAGGG |
| *NOS2* | AGGTCCAAATCTTGCCTGGG | ATCTGGAGGGGTAGGCTTGT |
| *Arginase 1* | GTCTGTGGGAAAAGCAAGCG | CACCAGGCTGATTCTTCCGT |
| *α-tubulin* | CCAGCGTGTCTGCTCAAAAC | ATTGCCCATCTGGACACCTG |

### Western blot analysis

After measuring the protein concentration using a BCA Protein Assay kit (Cat. 2329, Thermo Scientific), 50 μg of protein per sample was separated by sodium dodecyl sulphate-polyacrylamide gel electrophoresis (8% gel), transferred to a nitrocellulose membrane, and separately probed with each of the following antibodies: TLR4 (1:1000, Cat. A0007; ABclonal, USA), GFP (1:1000, Cat. 50430-2-AP; Proteintech), FLAG (1:1000, Cat. 14793S; Cell Signaling Technology), GAPDH (1:1000, Cat. 2118S; Cell Signaling Technology), β-actin (1:1000, Cat. 4970S; Cell Signaling Technology), Phospho-Akt (1:2000, Cat. 4970S; Cell Signaling Technology), Akt (1:1000, Cat. 9272S; Cell Signaling Technology), Phospho-FAK (1:1000, Cat. 8556T; Cell Signaling Technology), FAK (1:1000, Cat. 3285T; Cell Signaling Technology), Integrin β3 (1:1000, Cat. Ab179473; Abcam) and α-tubulin (1:5000, Cat. ab7291; Abcam). After overnight incubation, the membrane was washed with 0.25% Tween 20 in Tris-buffered saline, then probed with the corresponding secondary antibody conjugated with horseradish peroxidase (HRP). Signals were detected using the ChemiDoc XRS+ System (BIO-RAD, USA).

### Nuclear factor (NF)-kB transcription activity assay

THP-1 cells were treated with PBS or 50 μg/mL ANGPTL3 FBN domain for 24 h. Thereafter, cells were stimulated with 100 ng/mL lipopolysaccharide (LPS; Cat. L4391, Sigma, USA) for 15 min. Nuclear proteins were extracted using a Nuclear Extraction kit (Ab113474; Abcam, USA). After determining protein concentrations using a BCA kit, 5 µg protein samples were transferred to 96-well plate pre-coated with dsDNA of NF-kB provided in the Kit. After overnight incubation, samples were probed with NF-kB Ab for 1 hour followed by goat anti-rabbit HRP conjugate for 1 hour. Developer solution was added for colourisation and signal was determined by the absorbance at 450 nm.

### Migration assay

Cell migration assay was performed using the modified Boyden chamber with a pore size of 5 μm (Corning Incorp., USA) in 24-well plates. After count, 5 × 10^4^ cells suspended in 300 μl of RPMI 1640 medium were seeded in the upper compartment. In the meantime, 500 μl PBS containing 50 μg/mL ANGPTL3 FBN domain or PBS alone was added in the lower compartment. Cells were allowed to migrate for 6 hours at 37°c. The membranes were fixed with 4% PFA for 15 min and stained with DAPI. The migrated cells were pictured by a fluorescent microscope (ZEISS AXIO Observer Z1, Germany) and counted in five random fields using ImageJ.

### Peptide mass spectrometry (MS)

Protein extracts were digested with 1/25 trypsin (Promega) for 12 h at 37°C. The resultant peptides were reconstituted in 120 μL 0.5 M TEAB and treated with a tandem mass tag (TMT) sixplex Label Reagent kit (90068; Pierce). The peptides were then dissolved in solvent A (0.1% formic acid [FA] in H_2_O), loaded onto an Acclaim PepMap 100 C18 trap column (75 um × 2 cm; Dionex) using an Ultimate 3000 nanoUPLC system (Dionex), and eluted onto an Acclaim PepMap RSLC C18 analytical column (75 um × 25 cm; Dionex). The gradient of solvent B (0.1% FA in 80% acetonitrile [ACN]) increased from 2% to 10% over 6 min, climbed to 80% in 2 min, and then held at 80% for 4 min, all at a constant flow rate of 300 nL/min.

The peptides were subjected to nano-spray ionisation (NSI) followed by MS in a Q Exactive HFx mass spectrometer (Thermo Scientific) coupled online to the nanoUPLC system. Intact peptides were detected in the Orbitrap at a resolution of 70,000 (m/z scan range: 350–1500). A data-dependent procedure that alternated between one MS scan followed by 20 MS/MS scans was applied for the topN precursor ions above a threshold of 5E4 with 15 s dynamic exclusion. Peptides were selected and fragmented for MS/MS using 28% normalized collision energy (NCE); ion fragments were detected in the Orbitrap at a resolution of 17,500. Automatic gain control (AGC) was used to prevent overfilling of the ion trap; 3E6 ions were accumulated for generation of MS spectra and 1E5 ions for MS/MS spectra. The max injection times were 250 ms for MS scan and 100 ms for MS/MS scan. Liquid chromatography (LC)-MS was performed by Zhenhe Biotech (Zhengzhou, China).

### Proteome database search

The resulting raw peptide data were searched against the proteome database using Maxquant (v.1.5.2.8). Mass error was set to 10 ppm for precursor ions and 0.02 Da for fragment ions. Trypsin was selected for enzyme specificity and two missed cleavages were allowed. Carbamidomethylation on cysteine (Cys), a TMTsixplex tag on lysine (Lys), and peptide N-termini were specified as fixed modifications, and oxidation on methionine (Met) and a TMTsixplex tag on tyrosine (Tyr) were specified as variable modifications. The data were also searched against a decoy (reverse) database to estimate false discovery rate (FDR). The calculated results were revalued using an algorithm percolator, and peptide-spectrum match (PSM) scores with a p value < 0.05 were accepted. For quantitation, a protein had to have at least two unique peptides above identity. The protein ratio type was median, and the median normalisation method was used.

### MS analysis of protein phosphorylation

The peptides were digested as described above. For enrichment of phosphorylated peptides, the peptides were dissolved in 80% ACN/6% trifluoroacetic acid (TFA) and then incubated with IMAC-Ti4+ beads at room temperature with gentle shaking. The beads were washed once with 50% ACN/0.5% TFA/200 mM NaCl and once with 50% ACN/0.1% TFA. The bound peptides were eluted with 10% NH_4_OH and 80% ACN/2% FA. All eluted fractions were combined, vacuum-dried, and cleaned with C18 ZipTips (Millipore). Enriched phosphopeptides were dissolved in solvent A (0.1% FA in H_2_O), loaded onto an Acclaim PepMap 100 C18 trap column (75 um × 2 cm) using an Ultimate 3000 nanoUPLC system, and eluted onto an Acclaim PepMap RSLC C18 analytical column (75 um × 25 cm). The gradient of solvent B (0.1% FA in 80% ACN) increased from 2% to 10% over 6 min, 10% to 20% in 45 min (for phosphoproteome, 10% to 25% in 65 min), climbed to 80% in 2 min and then held at 80% for 4 min, all at a constant flow rate of 300 nL/min.

LC-MS/MS analysis was performed blindly by Zhenhe Biotech Company. The peptides were subjected to NSI followed by MS in a Q Exactive HFx mass spectrometer coupled online to the nanoUPLC system. Intact peptides were detected in the Orbitrap at a resolution of 70,000 (m/z scan range: 350–1500).

A data-dependent procedure that alternated between one MS scan followed by 20 phosphoproteome MS/MS scans was applied for the topN precursor ions above a threshold of 5E4 or 2.5E3 with 15 s dynamic exclusion. Peptides were selected and fragmented for MS/MS using 28% NCE; ion fragments were detected in the Orbitrap at a resolution of 17,500. AGC was used to prevent overfilling of the ion trap; 3E6 ions were accumulated for generation of MS spectra and 1E5 or 5E4 ions for MS/MS spectra. Max injection times were 250 ms for MS scan and 100 ms or 200 ms for MS/MS scan.

### Phosphoproteome database search

The resulting raw data were processed using MaxQuant with the integrated Andromeda search engine (v.1.5.2.8). Tandem mass spectra were searched against the mouse (UniProt) database concatenated with the reverse decoy database. Trypsin/P was specified as the cleavage enzyme, allowing up to four missing cleavages, five modifications per peptide, and five charges. Mass error was set to 10 ppm for precursor ions and 0.02 Da for fragment ions. Carbamidomethylation on Cys was specified as the fixed modification; oxidation on Met and phosphorylation on Tyr were specified as the variable modifications for the phosphoproteome. FDR thresholds for protein, peptide, and modification site were set at 1%. Minimum peptide length was set at 7. TMT-sixplex was selected as the quantification method. All other parameters were set to default values. Site localisation probability was set as > 0.75.

### Immunocytochemistry

Adherent THP-1 cells were cultured in confocal dishes (diameter: 35 mm, Corning Incorp., USA). After washing, cells were fixed with 4% paraformaldehyde for 20 min at room temperature. After blocking with 4% bovine serum albumin, cells were incubated with PE-conjugated anti-human integrin αvβ3 (1:200; Thermo Scientific) at 4°C overnight. After washing, DAPI was added and signals were observed under a fluorescent microscope (ZEISS AXIO Observer Z1, Germany).

### Histology

To investigate atherosclerotic lesions, heart tissues collected from mice and plaque dissected from patients were fixed in 4% paraformaldehyde. Tissue sections (5-µm thickness) were obtained from paraffin blocks using a rotary microtome. Atherosclerotic lesions in haematoxylin and eosin (H&E)-stained sections from mice were quantified using ImageJ software.

### Immunohistochemistry

Paraffin sections were heated at 60°C for 2 h, dewaxed in xylene, and dehydrated through a gradient alcohol series. After antigen retrieval and blocking of endogenous peroxidase and nonspecific staining with 3% H_2_O_2_ and 3% bovine serum albumin, mouse heart sections were incubated with rabbit anti-mouse CD68 polyclonal Ab (1:200, Cat. 28058-1-AP; Proteintech, USA), TLR4 (1:100, Cat. A0007; ABclonal, USA), rabbit anti-mouse anti-αSMA polyclonal Ab (1:200, ab5694; Abcam), goat anti-mouse polyclonal ANGPTL3 Ab (1:200, Cat. PA5-47127; Invitrogen, USA), or rabbit anti-FLAG polyclonal Ab (1:200, Cat. 20543-1-AP, Proteintech) overnight at 4°C.

Human plaque sections were probed with rabbit anti-mouse ANGPTL3 (1:50, Cat. 11964-1-AP, Proteintech) overnight. The next day, the slides were incubated with the corresponding HRP-conjugated secondary Ab for 30 min at 37°C. Finally, the sections were visualised by diaminobenzidine solution and counterstained with haematoxylin. In parallel, negative control was performed on the plaque section with the omission of anti-mouse ANGPTL3.

For double staining, paraffin sections were probed with rabbit anti-mouse CD68 polyclonal Ab (1:100, Cat. 28058-1-AP; Proteintech) and goat anti-mouse ANGPTL3 polyclonal Ab (1:50, Cat. PA5-47127; Invitrogen) overnight. After washing, they were incubated with goat anti-rabbit FITC (1:200; Abcam) and donkey anti-goat Alexa Fluor 647 (1:200; Abcam) for 45 min. The sections were studied under fluorescent microscopy (ZEISS AXIO Observer.Z1).

### Statistical analysis

For all experiments, unpaired, two-tailed Student’s tests and Mann-Whitney tests were used to compare the means between two groups. For more than two experimental groups, one-way analysis of variance with Dunnett’s multiple comparison test was applied. Significance was indicated by a two-tailed α-level of 0.05.

## References:

[1] Guo X, Asthana P, Gurung S, Zhang S, Wong SKK, Fallah S, et al. Regulation of age-associated insulin resistance by MT1-MMP-mediated cleavage of insulin receptor. Nat Commun. 2022;13(1):3749.

[2] Madhusudhan T, Ghosh S, Wang H, Dong W, Gupta D, Elwakiel A, et al. Podocyte Integrin-β(3) and Activated Protein C Coordinately Restrict RhoA Signaling and Ameliorate Diabetic Nephropathy. J Am Soc Nephrol. 2020;31(8):1762-80.

[3] Pang X, He X, Qiu Z, Zhang H, Xie R, Liu Z, et al. Targeting integrin pathways: mechanisms and advances in therapy. Signal Transduct Target Ther. 2023;8(1):1.

# Table S1. General characterization of *ApoE^-/-^* and *ApoE*^-/-^*Angptl3^-/-^ (*DKO) mice.

|  | *ApoE-/-* | *ApoE^-/-^Angptl3^-/-^* (DKO) |
| --- | --- | --- |
| Number | 4-10 | 11 |
| Plasma cholesterol (mmol/L) | 7.3 (1.3) | 4.7 (0.7) |
| Plasma triglyceride (mmol/L) | 0.75 (0.27) | 0.07 (0.09) |
| Body weight (g) | 26.8 (1.2) | 28.2 (2.9) |
| Fasting blood glucose (mmol/L) | 4.5 (0.5) | 5.9 (1.3) |
| White blood cells (×10^9^/L) | 14.3 (4.4) | 12.5 (4.5) |
| Lymphocytes (×10^9^/L) | 9.5 (4.1) | 9.0 (3.0) |
| Monocytes (×10^9^/L) | 0.38 (0.15) | 0.32 (0.12) |
| Granulocytes (×10^9^/L) | 4.4 (0.4) | 3.1 (1.6) |
| Red blood cells (×10^9^/L) | 8.5 (1.6) | 8.9 (0.5) |
| Hemoglobin (g/L) | 133.5 (21.4) | 129.5 (10.8) |
| Platelets (×10^9^/L) | 1215.3 (665.6) | 1350.4 (598.7) |

Data were expressed as mean (SD). Unpaired t-test or Mann-Whitney test was used to compare the means between two groups.

# Table S2. Circulating cytokine profiles in *ApoE^-/-^* and *ApoE*^-/-^*Angptl3^-/-^ (*DKO) mice.

| Cytokines in blood | *ApoE*^-/-^ | *ApoE*^-/-^*Angptl3^-/-^* (DKO) |
| --- | --- | --- |
| Number | 10 | 9 |
| Eotaxin (ng/mL) | 576.1 (287.3) | 261.2 (76.1)# |
| IFN-ϒ (ng/mL) | 3.9 (7.2) | 13.3 (18.1) |
| IL-1α (ng/mL) | 102.8 (95.9) | 88.8 (63.8) |
| IL-1β (ng/mL) | 21.5 (46.1) | 15.2 (18.4) |
| IL-2 (ng/mL) | 3.0 (6.2) | 12.1 (15.1) |
| IL-6 (ng/mL) | 16.2 (28.0) | 6.5 (6.1) |
| IL-10 (ng/mL) | 48.3 (78.8) | 7.9 (12.8) |
| IL-17 (ng/mL) | 10.0 (19.5) | 5.4 (3.9) |
| IP-10 (ng/mL) | 259.5 (131.1) | 235.3 (38.5) |
| MCP-1 (ng/mL) | 33.2 (68.7) | 61.7 (93.6) |
| MIP-1α (ng/mL) | 61.7 (93.6) | 111.7 (89.7) |
| M-CSF (ng/mL) | 131.5 (395.9) | 24.7 (23.2) |
| MIP-2 (ng/mL) | 115.7 (286.5) | 122.6 (133.9) |
| VEGF (ng/mL) | 6.8 (12.9) | 5.0 (3.8) |
| TNF-α (ng/mL) | 8.1 (17.8) | 14.2 (33.5) |

Plasma levels of cytokines and chemokines were determined by MILLIPLEX Multiplex assays using Luminex technology. Data were expressed as mean (SD). #p<0.05. Unpaired T-test or Mann-Whitney test was used to compare the means between two groups.

# Table S3. Intracellular cytokine profiles in THP-1 cells treated with FBN domain Angptl3.

| pg/mg | PBS | Angptl3 |
| --- | --- | --- |
| sCD40L | 17.7 (7.3) | 18.7 (7.5) |
| EGF | 0.58 (0.09) | 0.62 (0.07) |
| Eotaxin | 13.8 (0.7) | 15.5 (0.9)ǂ |
| FGF-2 | 3005.5 (552.3) | 3326.0 (747.2) |
| FLT-3L | 3.70 (1.72) | 4.21 (1.73) |
| Fractalkine | 26.4 (7.3) | 31.2 (7.1) |
| G-CSF | 0.40 (0.11) | 0.46 (0.14) |
| GM-CSF | 0.64 (1.14) | 0.72 (0.54) |
| GRO-α | 17.1 (3.5) | 22.3 (2.5)# |
| IFN-α2 | 3.40 (1.08) | 4.06 (0.75) |
| IFN-ϒ | 0.37 (0.10) | 0.39 (0.12) |
| IL-1α | 18.3 (7.9) | 25.9 (7.4) |
| IL-1β | 2241.4 (358.1) | 2702.6 (375.8)* |
| IL-1RA | 3737.2 (811.3) | 4232.6 (519.8) |
| IL-2 | 0.13 (0.06) | 0.21 (0.07) |
| IL-3 | 0.36 (0.54) | 0.35 (0.67) |
| IL-4 | 0.72 (0.11) | 0.79 (0.23) |
| IL-5 | 0.13 (0.02) | 0.15 (0.05) |
| IL-6 | 1.50 (0.39) | 1.89 (0.48) |
| IL-7 | 0.15 (0.07) | 0.21 (0.09) |
| IL-8 | 372.0 (142.9) | 565.3 (186.0) |
| IL-9 | 2.02 (0.26) | 2.23 (0.33) |
| IL-10 | 0.75 (0.15) | 0.82 (0.11) |
| IL-12(p40) | 60.8 (48.5) | 70.8 (63.7) |
| IL-12(p70) | 2.31 (0.78) | 2.85 (0.80) |
| IL-13 | 7.33 (1.87) | 8.01 (2.23) |
| IL-15 | 1.50 (0.94) | 1.55 (0.52) |
| IL-17A | 0.66 (0.39) | 0.77 (0.31) |
| IL-17E/IL-25 | 15.0 (2.9) | 17.4 (4.1) |
| IL-17F | 4.86 (1.36) | 6.30 (1.74) |
| IL-18 | 1.12 (0.40) | 1.18 (0.42) |
| IL-22 | 0.96 (0.25) | 1.00 (0.14) |
| IL-27 | 392.2 (59.4) | 515.7 (30.5)ǂ |
| IP-10 | 15.2 (4.1) | 23.3 (10.3) |
| MCP-1 | 17.2 (3.7) | 18.1 (3.5) |
| MCP-3 | 5.78 (1.90) | 6.43 (1.57) |
| M-CSF | 244.5 (43.7) | 270.3 (111.2)* |
| MDC | 22.7 (3.4) | 21.9 (2.0) |
| MIG | 3.72 (1.07) | 4.91 (0.93)* |
| MIP-1α | 1140.0 (226.9) | 1594.9 (396.0)* |
| MIP-1β | 16.1 (6.6) | 23.5 (6.8) |
| PDGF-AA | 1942.6 (605.6) | 2098.9 (529.0) |
| PDGF-AB/BB | 21.1 (7.6) | 22.8 (5.6) |
| RANTES | 1464.6 (342.8) | 1635.3 (229.6) |
| TGF-α | 0.89 (0.28) | 1.01 (0.27) |
| TNF-α | 18.6 (5.0) | 29.3 (6.2)# |
| TNF-β | 1.42 (0.53) | 1.72 (0.54) |
| VEGF-A | 10.9 (6.8) | 16.7 (7.6) |

THP-1 cells were exposed to 0 or 50 μg/ml FBN domain Angptl3 for 24 hours. After harvest, cells were lysed for protein concentration determination by BCA. Equal volume of cell extract was loaded to cytokine measurement by MILLIPLEX® MAP. Intracellular cytokine levels were normalized by protein concentration. Data were expressed as mean (SD). *p<0.05, #p<0.01, ǂp<0.001. Unpaired T-test or Mann-Whitney test was used to compare the means between two groups. N=7-8.

# Table S4. Released cytokine profiles in THP-1 cells treated with FBN domain Angptl3.

| Cytokines (pg/ml) | PBS | Angptl3 |
| --- | --- | --- |
| Number | 7 | 8 |
| sCD40L | 75.4 (6.1) | 83.0 (9.2) |
| EGF | 3.37 (1.22) | 3.10 (0.30) |
| Eotaxin | 20.9 (1.5) | 21.1 (1.0) |
| FGF-2 | 55.0 (9.8) | 60.7 (13.9) |
| FLT-3L | 18.1 (1.5) | 18.9 (2.1) |
| Fractalkine | 54.5 (11.5) | 45.6 (8.0) |
| G-CSF | 12.4 (3.8) | 12.6 (2.4) |
| GM-CSF | 0.05 (0.05) | 0.05 (0.05) |
| GRO-α | 258.2 (46.1) | 297.2 (46.0) |
| IFN-α2 | 7.96 (4.07) | 6.68 (0.85) |
| IFN-ϒ | 1.05 (0.30) | 0.93 (0.14) |
| IL-1α | 3.58 (1.66) | 3.23 (1.00) |
| IL-1β | 16.0 (2.1) | 20.0 (2.5)# |
| IL-1RA | 1930.6 (223.5) | 1945.6 (283.6) |
| IL-2 | 1.93 (0.34) | 2.00 (0.24) |
| IL-4 | 1.74 (0.29) | 1.77 (0.32) |
| IL-5 | 0.55 (0.30) | 0.48 (0.08) |
| IL-6 | 19.2 (2.6) | 19.8 (1.9) |
| IL-7 | 0.08 (0.04) | 0.08 (0.04) |
| IL-8 | 8387.9 (1621.9) | 9245.0 (1352.2) |
| IL-9 | 3.04 (0.23) | 2.85 (0.41) |
| IL-10 | 5.09 (1.60) | 4.96 (1.79) |
| IL-12(p40) | 9.76 (2.11) | 9.73 (3.10) |
| IL-12(p70) | 5.15 (1.44) | 5.12 (1.73) |
| IL-13 | 21.8 (4.0) | 22.2 (3.7) |
| IL-15 | 2.45 (0.95) | 2.21 (0.35) |
| IL-17A | 3.13 (0.79) | 3.47 (1.13) |
| IL-17E/IL-25 | 29.7 (4.7) | 29.6 (3.1) |
| IL-17F | 17.5 (10.2) | 1428 (3.0) |
| IL-18 | 0.83 (0.22) | 0.81 (0.20) |
| IL-22 | 5.85 (1.34) | 5.27 (0.99) |
| IL-27 | 412.0 (49.0) | 446.1 (42.4) |
| IP-10 | 87.0 (23.9) | 117.3 (46.3) |
| MCP-1 | 1215.8 (344.3) | 1043.8 (130.1) |
| MCP-3 | 74.2 (6.8) | 80.0 (8.1) |
| M-CSF | 41.3 (18.2) | 43.4 (14.9) |
| MDC | 168.8 (16.9) | 159.7 (12.9) |
| MIG | 21.7 (7.4) | 23.6 (4.5) |
| MIP-1α | 2348.7 (691.5) | 3140.0 (714.9)* |
| MIP-1β | 277.1(91.6) | 301.9 (44.11) |
| PDGF-AA | 9198.1 (1535.2) | 9134.9 (1569.2) |
| PDGF-AB/BB | 50.5 (7.1) | 56.2 (4.9) |
| RANTES | 4644.7 (711.5) | 4689.6 (850.2) |
| TGF-α | 6.80 (0.47) | 7.17 (1.11) |
| TNF-α | 98.4 (24.4) | 125.7 (22.8)* |
| TNF-β | 4.93 (1.22) | 4.93 (1.04) |
| VEGF-A | 18.0 (7.5) | 22.5 (7.7) |

Equal volume was taken to proceed cytokine measurement by MILLIPLEX® MAP. Data were expressed as mean (SD). *p<0.05, #p<0.01. Unpaired T-test or Mann-Whitney test was used to compare the means between two groups.

#
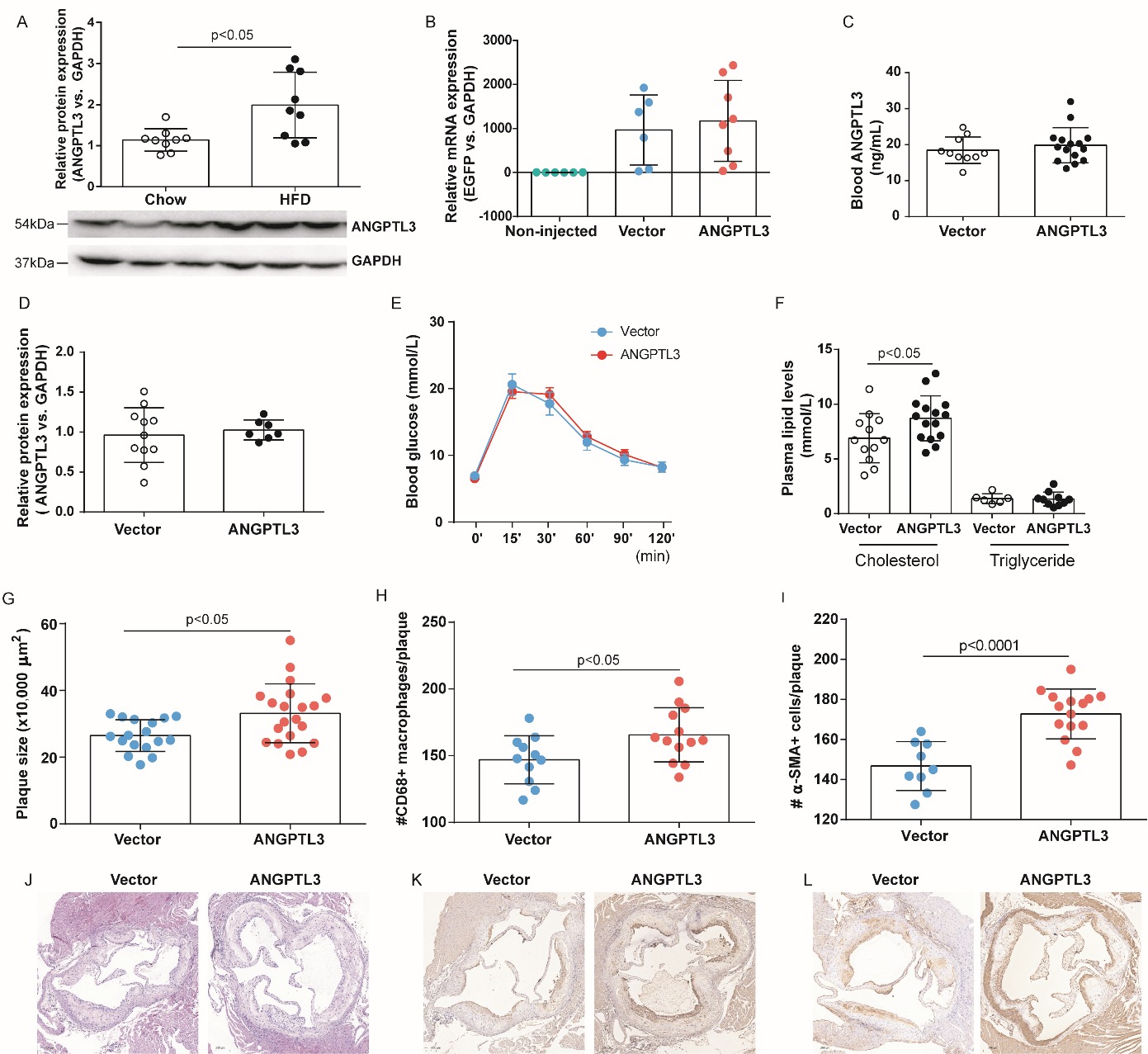
Fig. S1. Hepatic overexpression *Angptl3* increased plaque progression in hypercholesterolemia *Ldlr^-^*^/-^ mice. Male *Ldlr^-^*^/-^ mice at the age of 8 weeks were received AAV-mediated vector or *Angptl3* cDNA gene transfer. Immediately after gene transfer, mice were placed on high-fat diet (HFD) for 12 weeks. (A) Western blot analysis of hepatic Angptl3 expression in *Ldlr^-^*^/-^ mice on chow or HFD. N=9. (B) qPCR of EGFP expression in the liver. N=6–8. (C) Blood levels of Angptl3 by ELISA. N=10–15. (D) Hepatic expression of Angptl3 by western blot. N=7–11. (E) Glucose tolerance test. N=10–22. (F) Total cholesterol and triglyceride levels in the plasma. N=6–15. (G) H&E analysis of aorta. N=17–20. (H-I) Sections were stained with anti-mouse CD68 antibody or α-SMA antibody. The number of CD68^+^ macrophages or α-SMA^+^ smooth muscle cells were numerated in the plaque. N=9–15. (J) Representative H&E images, Scale bar: 200 μm; (K–L) Representative of CD68^+^ and α-SMA^+^ staining of cells in plaque, Scale Bar: 200 μm. Mann-Whitney test was used to compare total triglyceride levels between pooled controls and gene transferred mice (F). Except that, the rest of data were analysed by unpaired T-test (A–I).


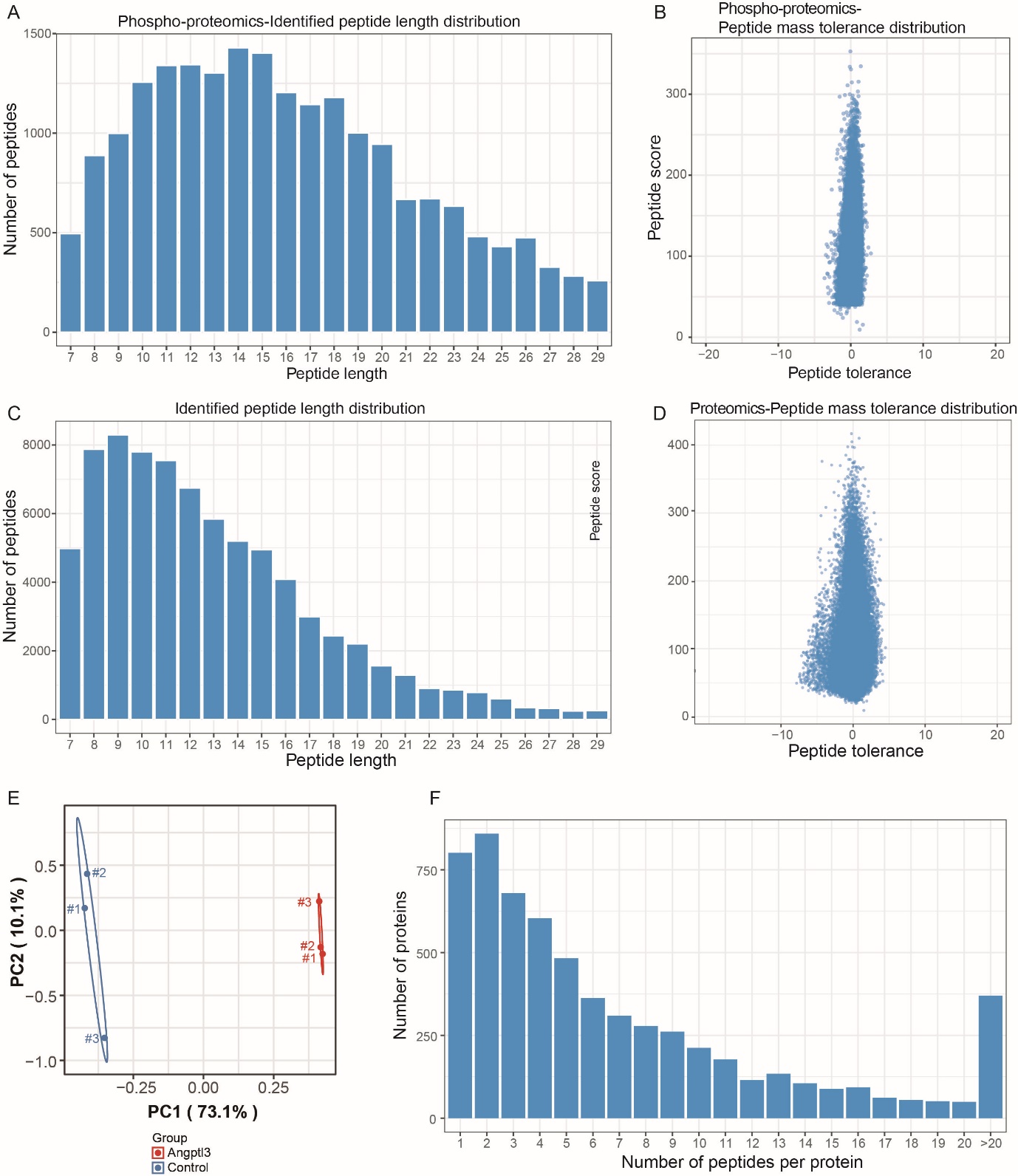


# Fig. S2. The overall GO analysis of ANGPTL3-treated THP-1 cells by phospho-proteomics mass spectrometry. THP-1 cells were treated without or with Angptl3 FBN domain for 15 minutes. Cells were subjected for phosphor-proteomics and proteomics. (A and B) Identified peptide length distribution and tolerance by phospho-proteomics. (C and D) Identified peptide length distribution and tolerance by proteomics. (E) PCA of proteomics. (F) Number of peptides per protein distribution.
